# Rational Design of Peptide-based Smart Hydrogels for Therapeutic Applications

**DOI:** 10.3389/fchem.2021.770102

**Published:** 2021-11-16

**Authors:** Saurav Das, Debapratim Das

**Affiliations:** Department of Chemistry, Indian Institute of Technology Guwahati, Guwahati, India

**Keywords:** peptide, hydrogel, drug delivery, tissue engineering, self-assembly, stimuli responsive

## Abstract

Peptide-based hydrogels have captivated remarkable attention in recent times and serve as an excellent platform for biomedical applications owing to the impressive amalgamation of unique properties such as biocompatibility, biodegradability, easily tunable hydrophilicity/hydrophobicity, modular incorporation of stimuli sensitivity and other functionalities, adjustable mechanical stiffness/rigidity and close mimicry to biological molecules. Putting all these on the same plate offers smart soft materials that can be used for tissue engineering, drug delivery, 3D bioprinting, wound healing to name a few. A plethora of work has been accomplished and a significant progress has been realized using these peptide-based platforms. However, designing hydrogelators with the desired functionalities and their self-assembled nanostructures is still highly serendipitous in nature and thus a roadmap providing guidelines toward designing and preparing these soft-materials and applying them for a desired goal is a pressing need of the hour. This review aims to provide a concise outline for that purpose and the design principles of peptide-based hydrogels along with their potential for biomedical applications are discussed with the help of selected recent reports.

## Introduction

Peptide-based soft materials have garnered great attention in the last couple of decades. Well-defined self-assemblies of peptides have been reported for as low as dipeptides to protein-like high molecular weight analogues(Gupta et al., 2020; Levin et al., 2020). The self-assembly process involves various supramolecular interactions and follows the hierarchical path in a concentration dependent fashion. One of the most common outcomes that lies at the top of this hierarchy is the formation of hydrogels(Wang et al., 2019). The emergence of molecular gels is programmed at the molecular level and is autonomously achieved in a bottom-up manner(Singh et al., 2017). Over the years, through a plethora of examples, the understanding of the mechanistic aspects of peptide hydrogels have been improved significantly(Dasgupta et al., 2013; Edwards-Gayle and Hamley, 2017; Singh et al., 2017). Based on the knowledge gathered in this area, it is now possible to design and prepare newer peptide assemblies with desired properties. A significant number of excellent reviews have been published in recent years that critically analysed the peptide aggregates and described models that help in designing new ones(Seow and Hauser, 2014; Singh et al., 2017; Sato et al., 2018).

**FIGURE 1:**
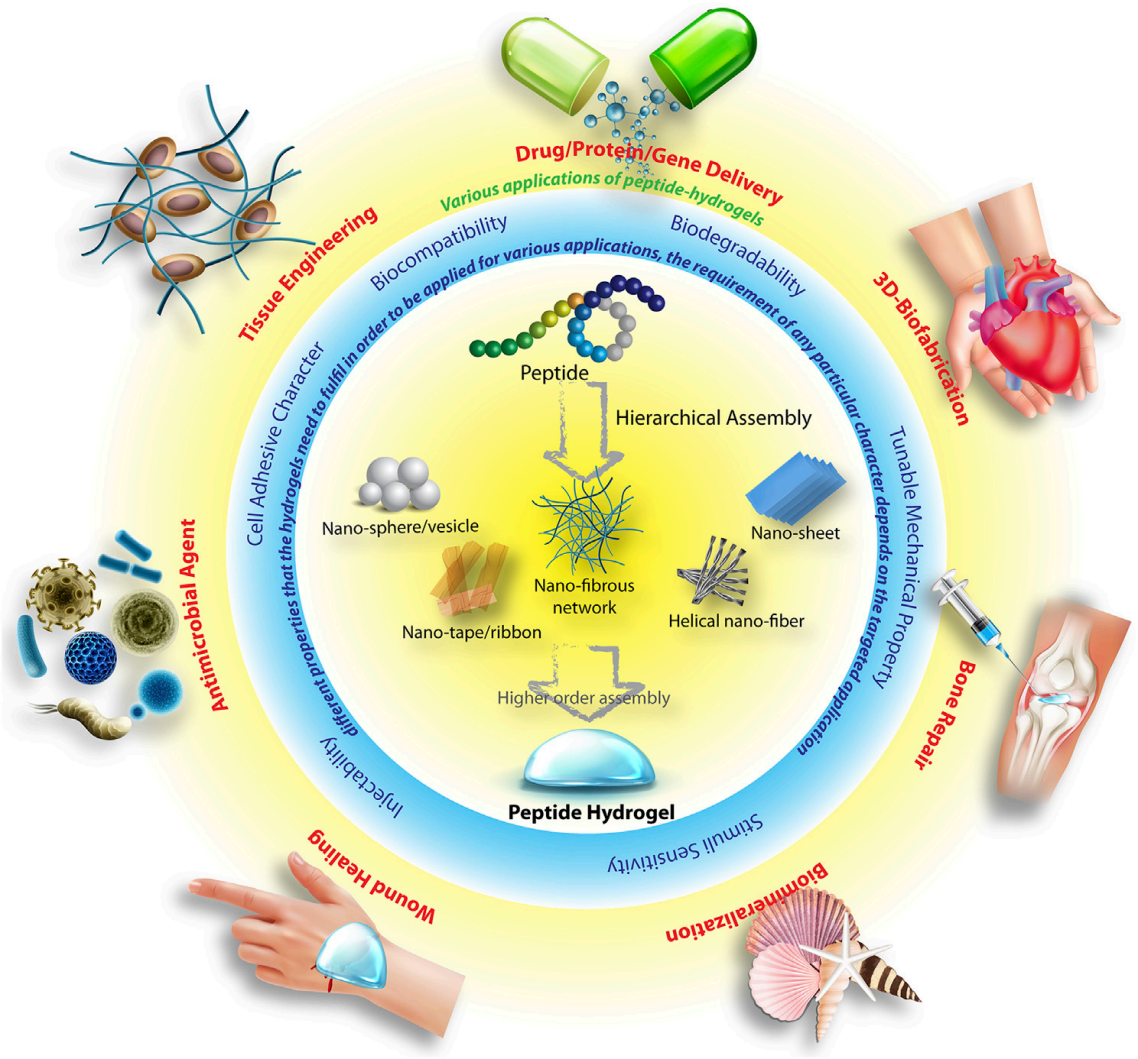
Schematic illustration of the hierarchical peptide self-assembly to different nano-structures and eventually to hydrogel, various important parameters which are essential for these hydrogels in order to use for various therapeutic applications. Parts of the figure are taken from reference (Dasgupta and Das, 2019) with permission. Copyright 2019 American Chemical Society.

The understanding of the supramolecular processes involved in the peptide assemblies also opened up the possibility of creating new generation of smart soft-materials with wide range of applications (Figure 1) (Ahmed et al., 2017; Lampel et al., 2018; Tian et al., 2018; Singha et al., 2019a; Singha et al., 2019b; Dasgupta and Das, 2019; Tarvirdipour et al., 2020). Amongst different types of applications of peptide hydrogels, their use in biomedical or therapeutic purposes holds the major share(Chen and Zou, 2019). The most important criteria a material needs to fulfil before it can be considered for therapeutic applications are, 1) biocompatibility, 2) biodegradability, 3) sustainability in aqueous biological fluids. Though there are several examples of peptides that are extremely toxic, in general, this class of molecules essentially fulfil the criteria of non-toxicity as well as biodegradability. Additionally, the ease of incorporation of stimuli sensitivity and tunability of mechanical properties of these materials make peptide based hydrogels inevitable choices for therapeutic and biomedical applications. Indeed, the last few decades have witnessed exhilarating advancement in the development of peptide hydrogels for a variety of therapeutic applications that range from, drug delivery (Kuang et al., 2014; Thota et al., 2016), tissue engineering (Kumar et al., 2017; Chowdhuri et al., 2020; Yang et al., 2020), wound healing (Xiao et al., 2016; Cui et al., 2020), biofabrication (Singh et al., 2008; Hedegaard et al., 2018) to name a few.

The extensive work in this area calls for the need to summarize the works charted so far and make an outline for the future directions. The current documentation aims to provide an overview of the present situation along with an analysis of the design principle for peptide hydrogels with therapeutic applications. The review encompasses the most relevant reports from the last decade and is exclusively focused on purely peptide based therapeutic hydrogels.

## Design Principle

### Design the Primary Sequence Based on Structural Aspects

Two decades back, the self-assembly of small molecules to form hydrogels were all serendipitous observations. However, over the years, fundamental understanding of the self-assembly processes has improved significantly. The hierarchical self-assembly model, in-depth understanding of the various non-covalent interactions, the concept of hydrophilic-lipophilic balance (HLB) (Mondal et al., 2014) provide a platform where one can successfully design and prepare hydrogels(Dasgupta et al., 2013). The structural aspects and balancing various supramolecular interactions in order to design a peptide-hydrogelator have been discussed in depth in several reports(Wang et al., 2016; Dasgupta and Das, 2019). Herein, we provide a short discussion on the structural aspects of the design criteria.

**FIGURE 2:**
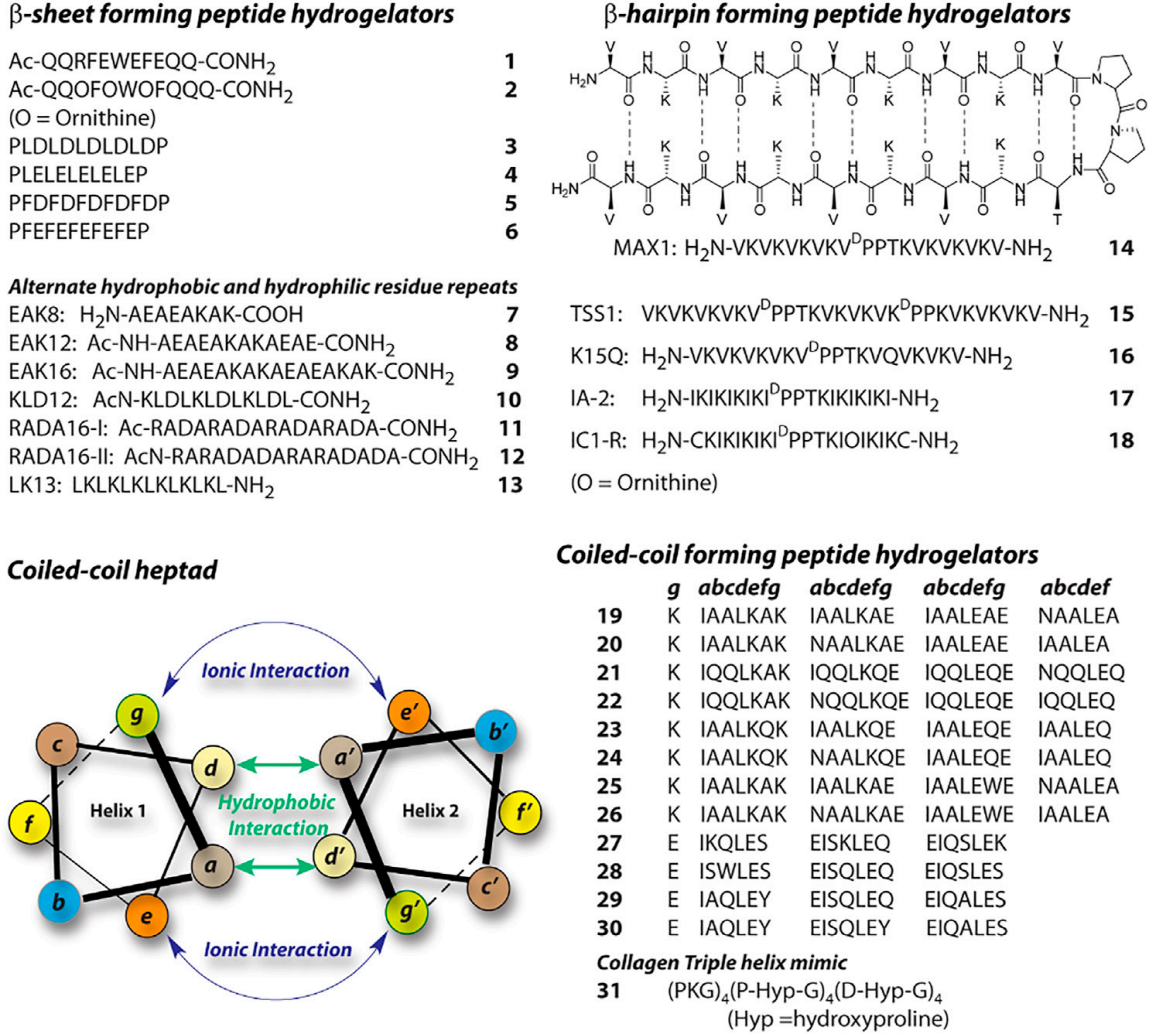
Sequences of representative peptide-hydrogelators classified according to the secondary structures.

Utilizing various supramolecular forces, proteins and peptides adopt different secondary structures like, β-pleated sheet, α-helix, coiled-coil, β-turn etc. Small peptides are also capable of forming secondary structure like arrangements. These structures further assemble in a hierarchical path to form higher order aggregates like hydrogels. A peptide’s probable secondary structure is determined by its primary sequence; hence, in order to build a peptide-based hydrogel, one must first identify the primary sequence that would eventually lead to a secondary structure capable of triggering hydrogelation. In this respect, β-pleated sheets are the most common choice. A plethora of β-sheet forming peptide hydrogelator are reported in literature. Boden and co-workers proposed a design principle for β-sheet type aggregates in solution, 1) cross-strand attractive forces between side chain functionalities, 2) the adjacent β-strands must recognize laterally for one dimensional self-assembly evading heterogeneously aggregated β-sheet structures, 3) strong adhesion of water to the surface of the sheets(Aggeli et al., 1997). Using this strategy, Ageli and co-workers synthesized one anionic (1) and one cationic (2) peptide (Figure 2). (Aggeli et al., 2003). The individual peptide show random coil structures in monomeric state. However, polyelectrolyte antiparallel β -sheet complexes (PECs) are formed on mixing aqueous solutions of 1 and 2 resulting in the spontaneous self-assembly of fibrillar networks and the production of nematic hydrogels. Periodic repeats of alternating hydrophilic and hydrophobic amino acids create discrete polar and nonpolar faces in the β-sheets. Thus, an amphiphilic character is developed within the peptide structure which allows higher order assemblies. This strategy is in-line with Boden’s design principle and have been used by several groups to create β-sheet forming peptides and their hydrogels. A list of some of those peptides are given in Figure 2 (3–13) (Zhang et al., 1993; Zhang et al., 1994; Zhang et al., 1995; Holmes et al., 2000; Kisiday et al., 2002; Rapaport et al., 2008; Geisler and Schneider, 2012).

Another interesting secondary structure, β-hairpin, is extensively exploited by Schneider et al. and later by other groups(Schneider et al., 2002; Li et al., 2017; Zhu et al., 2020). A Type-II’ β-turn is created between two strands of periodic repeats of alternating hydrophilic and hydrophobic amino acids. They used a tetrapeptide containing ^D^Pro-^L^Pro sequence flanked by two strands of (VL)_4_ (14). These peptides show stimuli driven folding to β-hairpin(Schneider et al., 2002). In the folded conformation, the resulting hairpin structures facilitate intermolecular hydrogen bonding as well as association of the hydrophobic faces, which lead to higher order self-assembly. There are several similar β-hairpin forming sequences reported and the sequences of some of these peptides are shown in Figure 2 (15–18) (Larsen et al., 2009; Rughani et al., 2009; Li et al., 2017; Zhu et al., 2020).

α-helical peptides are rarely featured in case of peptide hydrogel preparation. The primary reason behind that is the challenge to make these secondary structures assemble further. The hydrogen bonding associated with α-helices are typically intramolecular and thus these secondary structures are considered as discrete building blocks. α-helices can self-assemble through hydrophobic and ionic interactions between two helices when suitable amino acids are placed appropriately in the primary sequence. Peptide based hydrogels are created with coiled-coil motif which are basically supercoils formed by two or more α-helices (Xu et al., 2002). Typically, coiled-coils are designed as seven residue repeats (heptads) and the amino acids are designated as *a-g* (Figure 2)*.* In a typical heptad, the hydrophobic residues at *a* and *d* creates a hydrophobic core while the coiled-coil stability can be achieved through ionic interactions between the charged residues at *e* and *g*. Following this model, a series of 28 residue peptides (19–26) were reported by Woolfson (Ryadnov and Woolfson, 2003; Papapostolou et al., 2007; Banwell et al., 2009). These peptides co-assemble to form α-helical dimers with complementary sticky ends and proper modification in the peptide sequences lead to the formation of temperature sensitive hydrogels. Similarly following the helical wheel, Hartgerink reported four 21 residue peptides 27–30 (Dong et al., 2008). In these cases, to create hydrophobic patches, Ile (I) and Leu (L) are positioned at *a* and *d* respectively while positions *e* and *g* are filled with Glu (E). Importantly, the length and diameter of the fibers formed by the self-assembly of these peptides are governed by the residues present at *b*, *c*, and *f* positions. Hydrogels were formed by all four peptides under acidic pH where the glutamic acid residues are neutralized and thereby charge repulsion is avoided by the helices. Collagen triple helix mimicking peptides are another way to create coiled-coil peptide based hydrogels. Peptide 31 is one such example, which forms sticky-ended collagen like triple helices (Gribbon et al., 2008). These helices elongate and bundle into nano-fibers and subsequently form hydrogel at a higher concentration.

**FIGURE 3:**
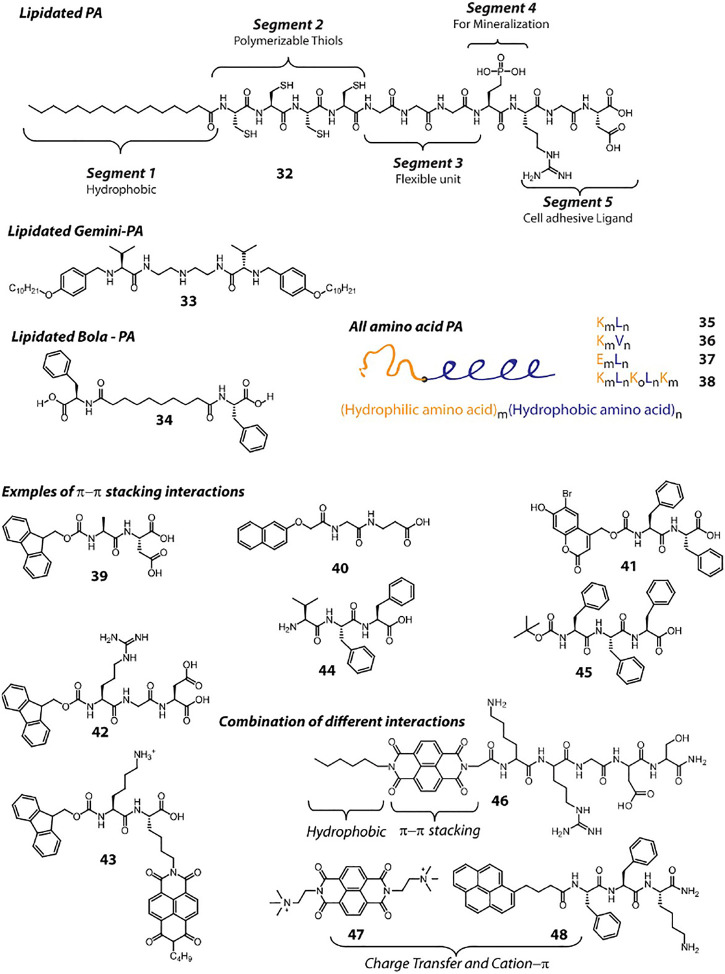
Chemical structures and sequences of representative peptide hydrogelators classified according to the supramolecular interactions involved in their self-assembly.

Hydrogen bonding is indigenously achieved by the peptides as the amide bonds provide both hydrogen bond donor and acceptor groups. However, placement of amino acid residues play key role toward hydrogen bonding, which affect the secondary structure formation and consequently to the higher order aggregation. π−π stacking and hydrophobic interaction also play crucial role to direct the path of aggregation. Hydrophobic interaction can be achieved by attaching alkyl chains to the peptide molecules. Lipidated peptide amphiphiles (PA) are common hydrogelator and the design principle for this class can be explained with the help of 32 (Figure 3), a classic example reported by Stupp et al. In this case, five different segments are incorporated within the PA as per the requirement for the targeted bio-mineralization application. Importantly, the attachment of the lipid group provides the required hydrophobicity to the molecule to attain appropriate HLB. Appropriate HLB is essential toward the self-assembly of the peptide and allows 32 to form cylindrical micelle and consequent hydrogelation. Lipidated peptides can be of different types, for example, gemini (33) or bola (34) amphiphiles and many such hydrogelators are already reported in literature (Ray et al., 2007). A hydrophilic sequence attached to a hydrophobic sequence lead to the formation of ‘all amino acid PA’s (35–38) and can also provide sufficient amphiphilicity toward self-assembly and hydrogelation (Holowka et al., 2005; Li and Deming, 2010). In these cases, amino acid stretches can be arranged as block co-polypeptides (Nowak et al., 2002). Peptides 7–18 also create amphiphilicity within the molecule as two different faces are generated during the folding, one with hydrophilic character and another with hydrophobic. All these peptides also fall in the “all amino acid PA” category.

The self-assembly process is primarily driven by thermodynamics. However, kinetics also play a critical role in structural modulation and function integration. The kinetic and thermodynamic aspects of peptide self-assemblies have been discussed in detail by Yan’s group in their review article (Wang et al., 2016). Three major energy contributions come from the hydrogen bonding, hydrophobic interactions, and electrostatic interactions (Cui et al., 2010). While the first two are attractive in nature and promote the assembly, the electrostatic forces could be dissociative. Therefore, a subtle balance is essential in the design of the peptide in order to facilitate the assembly process. The resulting structure, shape, size of the assembly depend on this balance. The HLB of peptides is crucial in determining the aggregation pathway. The thermal stability of a peptide secondary structure can be enhanced when a lipid group is attached to the peptide (Yu et al., 1996; Hamley, 2011). For example, a known β-sheet-forming peptide (KTVIIE) have shown higher thermal stability of the self-assembly when an alkyl chain is attached to it without affecting the final morphology (Meijer et al., 2007).

A common approach to create small peptide based hydrogelators is to attach aromatic groups, which provide π−π stacking interaction. To achieve that, chemists rely on groups like, Fmoc, Napthalene, Pyrene, Naphthalenediimide (NDI), Anthracene etc. Attaching these groups at the N-terminal is a common practice. Even protected (with these groups) amino acids (Fmoc-Phe, Fmoc-Tyr), di or tri peptides (39–43) can assemble under appropriate conditions and form hydrogel (Vegners et al., 1995; Yang and Xu, 2004; Yang et al., 2006; Orbach et al., 2009; Sutton et al., 2009; Shao and Parquette, 2010; Ikeda et al., 2011). The attachment of these π-rings can be done at the side chain functionality as well (43). Along with π−π stacking, these fused π-systems also provide hydrophobic character and thus these peptides can also be considered as PAs. Several detailed reviews discussing about these peptides can be found in literature (Fleming and Ulijn, 2014; Hu et al., 2020). Moreover, the aromatic rings present in Phe, Tyr or Trp can also be rationally utilized to get sufficient intermolecular π−π stacking that lead to hydrogelation (44, 45) (Palui et al., 2009; Marchesan et al., 2012).

In addition to the π−π stacking interaction, design of molecules can also be done by incorporating combinations of different interactions. The π—electron systems can be attached to peptides in addition to other hydrophobic groups (a C_6_ chain and NDI in case of 46) to provide additional stability to the aggregates (Singha et al., 2017). Recently, we have demonstrated the combination of charge transfer (CT) and cation—π interactions between two molecules that lead to the formation of a self-healing hydrogel. In this case, the NDI unit of 47 being electron deficient, accepts electron pool from the Pyrene ring of 48 and thereby creates a CT-complex (Pramanik et al., 2019). The complex is further stabilized through cation—π interaction between the quaternary ammonium group of 47 and the π cloud of 48.

The path of the self-assembly of peptides are dictated by the polarity of the medium. Recently we have shown the solvent polarity dependent morphogenesis of a bola-peptide amphiphile. The PA adopts helical nano-fibrous structure in apolar or low polar solvent (Ahmed et al., 2017; Ahmed et al., 2018). However, with increase in solvent polarity, the morphology changes through nano-spheres to nano-ring. For this PA, in non-polar medium, aggregation is kinetically controlled but in polar solvent, the assembly is governed by the thermodynamic parameters (Ahmed et al., 2017). For a molecule to form gel, the solubility in the gelation solvent is an important parameter. A general rule is that the molecule must be soluble but should not be highly soluble in the medium/solvent. In some cases, addition of small amount of water-miscible organic solvents are used to solubilize the gelator and addition of aqueous medium decreases the solubility and enhances the aggregation propensity. However, these gels certainly cannot be used for *in-vivo* therapeutic applications. Since, the use of organic solvents is not an option for therapeutic hydrogels, the major influence comes from the pH and the ionic strength of the medium. While designing a peptide based gelator, one has to take care of the polar groups and the pH of the targeted working medium. For pure aqueous gel formation, in cases of anionic peptides, the peptides are solubilized in basic buffer and the pH of the system is adjusted to lower pH in order to reduce the solubility of the gelator so that the aggregation process can be achieved. The reverse is true for cationic peptides (Dasgupta and Das, 2019). Adjustment of the pH of the medium is a common practice. However, pH adjustment method also dictates the aggregation pattern as reported by Adams. Rapid adjustment of pH by addition of acids produces irreproducible results while a slow and kinetically controlled acidification via the hydrolysis of lactones results in reproducible and uniform aggregated structures (Adams et al., 2009; Draper and Adams, 2019).

The possibility of controlling/manipulating the soft-materials using stimuli are essential for most of the therapeutic applications. Hence while designing a peptide hydrogelator for therapeutic application, one has to incorporate the desired stimuli responsive group within the sequence. A detailed discussion on this issue is provided in the section *Stimuli-Responsive Hydrogels*.

### Cytocompatibility

One of the prime requisite for a therapeutic material is that it should be non-toxic and biodegradable. In this regard, peptide based systems are always advantageous as the main building blocks are amino acids. The toxicity of a peptide in general depends on its physicochemical properties like amino acid sequence, charge, length, amphipathicity, hydrophobicity, and the secondary structure it adopts. Manipulating these properties may be helpful to minimize the toxicity of peptides. However, for peptide-based hydrogels, these changes should not come as a compromise to the self-assembling behaviour. Unfortunately, peptide materials exhibiting detrimental and toxic nature rarely surface in literature reports and consequently identifying and rationalising cytotoxic peptide sequences becomes extremely cumbersome. However, effective models and softwares are available on toxicity prediction for short peptides. Since these soft-materials are designed for therapeutic applications, biodegradability of these systems is another important factor that needs attention during the design stage. The biomimetic nature of peptides, in general, not only renders them cytocompatible but also makes them susceptible to degradation by enzymes and microbes facilitating easy removal of the peptide based therapeutics from the body once their purpose is served. Furthermore, like toxicity, biodegradability prediction models are also available in literature (Pavan and Worth, 2008). These platforms could be used as a good starting point for designing possible non-toxic and biodegradable sequences. There are several ways to improve the therapeutic index of peptides and a detailed discussion on this issue can be found in the review by Raghava et al. (Gupta et at., 2015).

### Mechanical Property

The mechanical property of a hydrogel dictates its applicability for a particular purpose. An in-depth understanding of the mechanical properties is essential in order to determine the suitability of the hydrogel for a targeted therapeutic application. The mechanical property of the hydrogel is crucial to uphold the scaffold stability to bear loads. On the other hand, at microscopic level, the mechanical signal affects the cell activities. Matrix (the hydrogel) stiffness affect the cell spreading, migration, proliferation, and differentiation (Wen et al., 2014). There are several techniques that are used to estimate and understand the mechanical properties of hydrogels. Some of the common tests are, extensiometry, bulge test, indentation test, compression test etc. However, for supramolecular hydrogels, the most common way to determining the mechanical property is rheology.

In fact, the rheological property of a hydrogel become extremely crucial when it is required for an application inside human body, eg, localized drug delivery or biomedical implants. The most common approach for these purposes is to inject liquid precursors at the desired site, which eventually undergo sol to gel transition (via chemical or physical cross-linking) at the site of injection. However, this approach raises several uncertainty including, possible wash out by the body fluid, incomplete cross-linking resulting into possible toxicity etc. For that purpose, it is desired to get hydrogels with shear-thinning property (Sathaye et al., 2015). A shear thinning hydrogel displays progressive fall in viscosity and exhibits fluid-like flow properties once a shear of large amplitude is applied. Upon removal of the shear, the fluid recovers back to the original hydrogel form in a time dependent manner (Self-healing). The shear thinning property arises on account of transient disruption of dynamic non-covalent interactions (e.g., hydrogen bonding, electrostatic interactions, etc.), that uphold the hydrogel network structure together, under the influence of an applied shear. Removal of shear causes reassembly of the networks and consequent recovery of the hydrogel state (Guvendiren et al., 2012). Peptide based hydrogels display extensive non-covalent interactions driven nanofibrillation and thus are the utmost candidate for designing shear thinning hydrogels. These hydrogels can be pre-formed in a syringe or catheter and injected at a desired site of body and re-healed into a percolated solid gel after injection. These gels are termed as injectable gels (Dowari et al., 2020). Such hydrogels can be pre-formed in presence of the payload (drug, protein, gene, and growth factors etc.) and thereby can encapsulate the payload within the physical cross-links of the hydrogel. Thereby gel entrapped payload can be delivered at the site where it remained encapsulated within the hydrogel network and can be released with the help of a stimuli or specific molecules present in the body fluid or at the site.

**Scheme 1:**
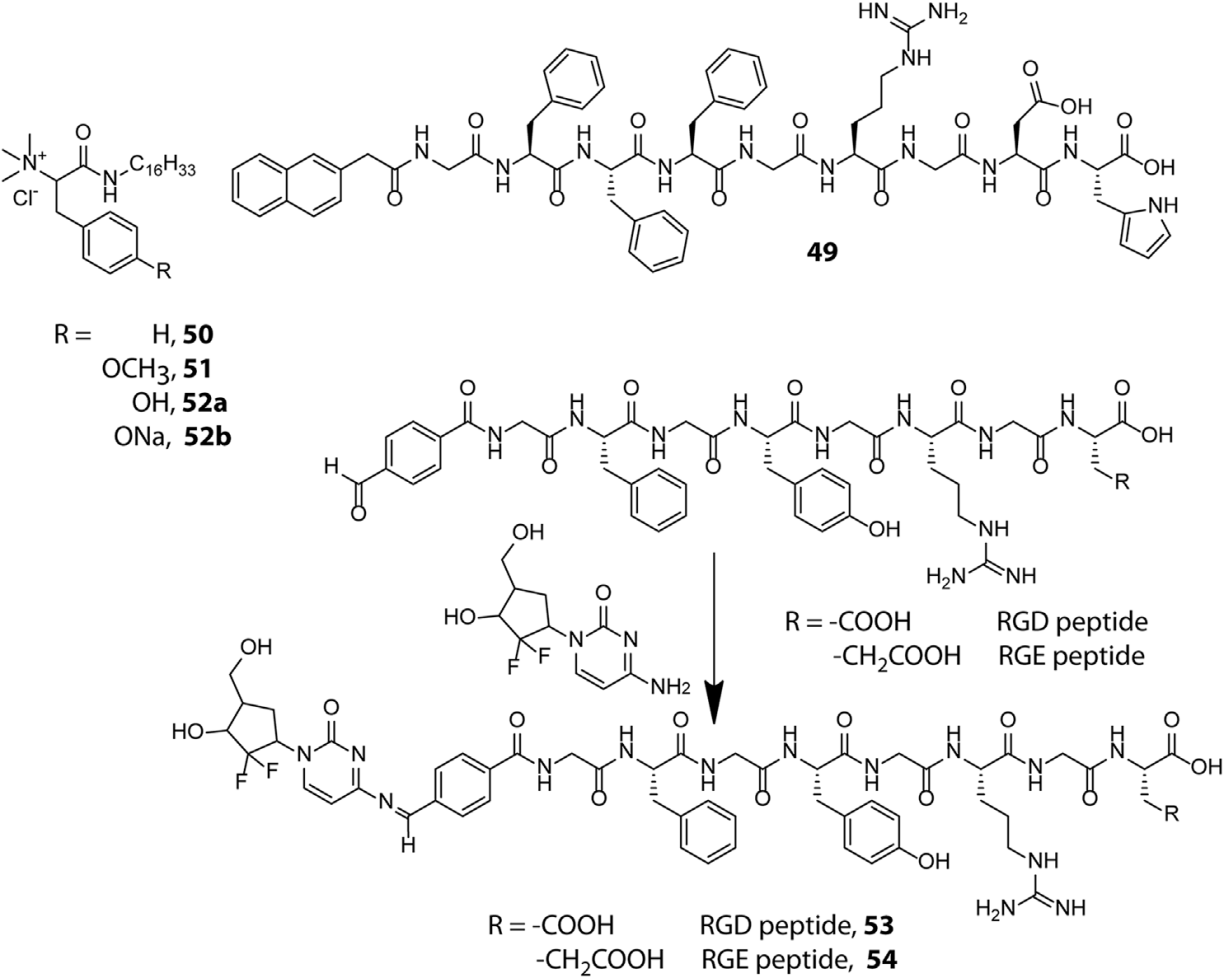
Chemical structures of exemplary pH responsive peptide hydrogelators used for therapeutic applications.

The general method applied to understand the mechanical properties of a hydrogel is to measure the rheology where storage modulus (G′) and loss modulus (G″) and loss factor tan (δ) are recorded as functions of oscillatory frequency, oscillatory strain and time (Yan and Pochan, 2010). The G’ and G” qualitatively represent the material stiffness and liquid like flow properties. These measurements help in understanding the linear viscoelastic regions, relaxation time scale and gelation kinetics. Overall, on the basis of the mechanical stiffness, hydrogels can be classified into three different categories, 1) weak gel (0.1–1 kPa), 2) moderate gel (8–17 kPa) and 3) strong gel (34 kPa) (Scheme 1). (Li et al., 2018) Though stiffness is not the only parameter to be looked at, the application of the hydrogel can be selected based on their mechanical property. Thus, standard rheological measurements provide sufficient information based on which the applicability of the hydrogels can be decided. Subsequently, fine-tuning of the rheological properties can be achieved through modification of the gelator molecule or manipulating the hydrogel through addition of appropriate additives. Modulating the rheological property to achieve desired applicability can be discussed with the help of a report by Adler-Abramovich group (Ghosh et al., 2017). The rigidity of the hydrogel formed by Fmoc-FF is modulated with appropriate amount of another non-gelling building block, Fmoc-R. The co-assembly of these two molecules lead to a fitting mechanical property of the hydrogel in presence of hydroxyapatite (HAP), a well-known inorganic material that helps in cellular growth of human osteoblasts. Fibroblast cells could efficiently proliferate in these co-assembled hydrogel matrices owing to appropriate rigidity of the gel, presence of HAP and cell-adhesive units in the form of Fmoc-R.

### Cell-Adhesion

For tissue engineering as well as localized drug delivery applications, it is important that the hydrogel can adhere to the tissues. Attachment of cells to the extracellular matrix (ECM) is a prerequisite for various important cell functions like, cell proliferation and cell migration (Ding et al., 2020). The cell-surface receptors bind with the cell-adhesive domains available and these interactions play crucial role toward the tissue development, organization and maintenance. Thus, to utilize hydrogels as an artificial ECM, ability to adhere to cells is essential. On the other hand, for use as a drug delivery platform, the drug loaded hydrogels need to stick to the tissue so that a prolonged and sustained release can be achieved. To achieve the tissue/cell adhesion, various strategies are in use.

The most commonly used methodology is to incorporate peptide sequences derived from ECM-protein capable of cellular adhesion (Dowari et al., 2018), These peptides are called cell-adhesive peptides (CAP). Short peptide sequences from six ECM proteins, namely, laminin, bone sialoprotein, fibronectin, collagen, vitronectin, and elastin are used as adhesive agents (Zhu and Marchant, 2011). One fundamental requirement for a CAP to be successful is that it should retain its biological specificity when taken out from its native protein structure. Short peptide sequences derived from these proteins are advantageous over using the entire protein sequence as they are relatively stable, easily tunable to fit with the system and can be synthesized on a large scale.

In this regard, the most common sequence used is RGD, derived from fibronectin, an intergrin-binding peptide. Other CAP sequences that are also used to achieve cell-adhesion property are, VAPG (elastin) YIGSR, KQAGDV and REDV (fibronectin), LGTIPG, IKVAV, PDGSR, LRE, LRGDN and IKLLI (lamnin), and DGEA and GFOGER (Collagen). These CAPs are incorporated into peptide hydrogels through chemical conjugation or as an additive to co-assemble.

Recent studies have revealed that stereoselective interaction between chiral peptide assemblies and biologically relevant entities such as cells, proteins, etc. greatly influence the degree of cell adhesion and proliferation on or within the assemblies. As chirality is a distinguishing feature of biological systems, different proteins and cells show preferential interactions with one particular type of enantiomeric peptide hydrogel over the other. Chiral peptide hydrogelators exhibit amplified supramolecular chirality upon formation of self-assembled structures and moreover, peptide hydrogels derived from L-amino acids display more pronounced cell adhesion, proliferation and differentiation propensity, as compared to D-type peptide hydrogelators (Liu et al., 2014; Dou et al., 2019). The chiral recognition feature of peptide-based hydrogels, thus, serve as a key design element that can be easily tweaked to tune the cell adhesive properties of 3D hydrogel matrices.

Another approach that can be used to improve cell-adhesion is to use biopolymers with cell-adhesion property. Proteins like collagen or collagen derived gelatin can be used for enhancing the adhesive property. Polysaccharides like hyaluronic acid, dextran, chitosan, heparin, and alginates can also be used in order to enhance adhesion property of a peptide-based hydrogel. These natural polymers can form hydrogels themselves under various conditions and when co-assembled with short peptide based hydrogelators, the network formed thereby provide an adequate environment for the cells to adhere and proliferate. Even biodegradable synthetic polymer like polyethylene glycols can also be conjugated or used as an additive to increase the adhesive property of peptide-hydrogels. However, it is to be noted that one has to sacrifice the specificity by using these synthetic polymers.

## Stimuli-Responsive Hydrogels

The nature and extent of the non-covalent interactions and thereby the mechanical strength and robustness of the hydrogels is primarily dictated by factors such as pH, temperature and ionic strength of the gelation medium. Strategic incorporation of destabilizing functions in the hydrogel framework that may disturb the structural integrity of the hydrogel in response to a specific environmental condition serves as the fundamental strategy for designing stimuli responsive hydrogels. Unhealthy disease bearing tissues and cells display stringently different physiological parameters as compared to healthy tissues, e.g., tumour tissue microenvironment exhibits low pH, low oxygen content (hypoxia), elevated levels of reactive oxygen species (ROS), over-expressed receptors and hydrolytic enzymes (Cairns et al., 2011). These distinctive disease specific stimulants can be used to our advantage for engineering stimuli responsive hydrogels. Some selected examples portraying a distinct array of strategies employed for realizing stimuli responsiveness are discussed in the succeeding sections.

### pH Responsive

Biological fluids exhibit wide variations in pH ranging from 1–3 in stomach to 8 in liver and pancreas. The tumour microenvironment (TME) shows a slightly acidic pH of 6.5–6.8 on account of higher rate of glycolysis and malfunctioning lymphatic drainage system which leads to excessive production and accumulation of lactate in the tumour extracellular matrix (Cairns et al., 2011). This pH difference of ∼1 unit between the TME and normal tissues (∼7.4) has been exploited for designing pH sensitive carriers for cancer theranostics. pH gated manipulation of charged interaction between pH sensitive amino acids such as lysine, arginine, histidine, aspartic acid, and glutamic acid in peptide gelator backbone has been one of the most popular approach to control gelation propensity of peptide hydrogels. Schneider et al. using their β-hairpin peptides exemplified the important role played by pH on the attainment of secondary structural conformations critical for hydrogelation. MAX1 (14, Figure 2 and Table 1) demonstrated pH induced switching of sol-gel properties (Schneider et al., 2002). Partial deprotonation of some of the Lys residues under alkaline conditions instigates the MAX1 peptide to undergo intramolecular folding and adopt a β-hairpin conformation and the hydrogel is formed. Exposure to acidic environment leads to intra-strand charge repulsion between the protonated lysine residues and induces unfolding of the hairpins resulting in the dissolution of the hydrogel. However, the high pH (∼9) required for hydrogelation of MAX1 makes it particularly unsuitable for biomedical and especially for *in-vivo* applications. Screening the charged lysine residues of MAX1 by increasing the ionic strength of the gelation medium was found to be a much conducive strategy to trigger hydrogelation at physiological pH (∼7.4) (Kretsinger et al., 2005). MAX1 hydrogel prepared in buffered (pH 7.4) saline solution (150 mM NaCl, 2 wt% peptide) demonstrated excellent cytocompatibility, cell adhesion and proliferation for fibroblast cells.

**TABLE 1:** List of different stimuli responsive peptide hydrogelators.

Stimuli	Peptide sequence (short name, number)	Hydrogel degradation mechanism	Ref
pH	VKVKVKVKV^D^PPTKVKVKVKV-NH_2_ (MAX1, 14)	β-hairpin unfolding at acidic pH	Schneider et al. (2002); Kretsinger et al. (2005)
	Nap-GFFYGRGDH (49)	Acidic pH mediated protonation of histidine leading to hydrogel dissolution	Mei et al. (2019)
	(CH_3_)_3_N^+^-(Tyr-O^-^Na^+^)-CONH-C_16_H_33_ (50–52)	Destabilization of equilibrium between electrostatically coupled tyrosine and tyrosinate residues at acidic pH	Shome et al. (2008)
	Gem-FBA–GFFYGRGD (53) and Gem-FBA–GFFYGRGE (54)	Hydrolytic cleavage of imine linked gemcitabine at acidic pH	Ren et al. (2014)
	(Gem: Gemcitabine, FBA: 4-formylbenzoic acid)		
Redox	PBMA-GA-FFY (55)	GSH triggered removal of self-immolative PBMA group	Zheng et al. (2018)
	(PBMA: 4-(2-Pyridinyldithio)benzenemethanol, GA: Glutaric anhydride)		
	Py-KC (Py: Pyrene) (59)	GSH triggered disulphide cleavage	Singha et al. (2019c)
	PhSe(CH_2_)_3_COFFYEE (61)	H_2_O_2_ mediated oxidation of hydrophobic selenide to hydrophilic selenoxide	Miao et al. (2013)
	Fc-F (63) (Fc: Ferrocene)	H_2_O_2_ mediated oxidation of ferrocene to ferrocenium ion leading to loss of aromatic interaction between ferrocene and phenylalanine residues	Sun et al. (2013)
	Nap-GFFYGD(Thi) (64) (Thi: Thiazolidinone)	H_2_O_2_ triggered cleavage of thiazolidinone group	Ren et al. (2017)
Enzyme	Nap-FFK-Olsalazine (66) (Nap: Naphthalene)	Azo reductase catalysed reduction of the azo linkage triggers gel-sol transition with concomitant release of mesalazine	Li et al. (2010)
	Ac-YYYY-OMe (67)	Tyrosine catalysed conversion of tyrosine to quinone leading to disruption of aromatic π−π stacking interactions	Gao et al. (2011)
	Ac-I_3_SLKG-NH_2_ (69)	MMP2 catalysed S-L peptide bond digestion	Chen et al. (2017)
	C_12_GG-RGDR-PLGVR-VVV (RBA-1,70)	MMP2 triggered scission of G-V peptide linkage	Chakroun et al. (2020)
	IKIKIKIK-I^D^PPT-KIOIKIKI-NH_2_ (IA-2, 17)	GOx mediated conversion of glucose to gluconic acid leading to hydrogel dissolution at acidic pH	Li et al. (2017)
	CH_3_CO-OVTVOVDVOVTVOVDV-NH_2_ (F-4, 71)		Fu et al. (2018)
	BPmoc-FF (72) (BPmoc: p -borono-phenylmethoxycarbonyl)	GOx mediated conversion of glucose to H_2_O_2_ leading to oxidative cleavage of BPmoc group	Ikeda et al. (2011)

Incorporation of drug molecules often alters the mechanical properties and stimuli responsiveness of the hydrogel, resulting in poor and unsatisfactory release kinetics. Zhong et al. circumvented this issue by adopting a drug-reinforced hydrogelation strategy wherein electrostatic interaction between the aspartate residue of the peptide gelator, Nap-GFFYGRGDH (49, Scheme 1), and the chemotherapeutic drug, doxorubicin, lowered the MGC (Minimum Gelation Concentration) of the peptide and boosted the self-assembly process as well as the pH responsive attributes of the hydrogel (Mei et al., 2019). The RGD sequence conferred the hydrogel with tumour adhesion properties and the ionisable histidine group at the N-terminus of the peptide imparted pH responsiveness to the hydrogel. The sheer-thinning co-assembled hydrogel displayed a two-step release kinetics wherein the protonation of histidine residues at mild acidic pH (∼6.5) of the tumour microenvironment triggered partial disentanglement of the nanofibers which later were internalised into the tumour cells through integrin receptor mediated endocytosis and completely disrupted by the endosomal pH (∼5.5) to release the active drug for anti-cancer activity. *In vitro* as well as *in vivo* experiments revealed that the hydrogel displayed good cytocompatibility, prolonged release kinetics and effectively inhibited the proliferation of non-small-cell lung cancer cells. Identifying and incorporating favourable interactions between the drug and hydrogelator molecules may thus prove to be an effective strategy to improve the overall efficacy of drug-peptide hybrid hydrogels for therapeutic applications.

Amphiphilic peptides constitute another important class of peptide gelators whose gelation propensity is primarily dictated by their HLB. Amphiphilic peptides containing pH sensitive amino acids in the head group provide the gelator molecules with pH switchable amphiphilicity and present a modular foundation for designing pH sensitive hydrogels. In this direction, Das et al. prepared a library of structurally analogous L-phenylalanine and L-tyrosine based amphiphilic peptides to study their structural implications on hydrogelation (50–52) (Shome et al., 2008). All analogues lacking a quaternary ammonium head group turned out to be non-gelators owing to the lack of amphiphilic character needed for hydrogelation. Also, the cationic head group bearing tyrosine based amphiphile (52a) failed to form hydrogel whereas the comparatively less hydrophilic phenylalanine (50) and methylated tyrosine (51) based analogues efficiently gelated water to form pH irresponsive hydrogels, signifying the importance of a proper HLB for realizing hydrogel formation. The sodium salt (52b), however, formed a pH sensitive hydrogel owing to electrostatic interaction between tyrosine and tyrosinate residues which exist in a pH dependent equilibrium mixture in aqueous medium. At acidic pH (pH ≤ 5.5), the sodium salt was protonated and the equilibrium was destabilized leading to the disintegration of the hydrogel network into an insoluble precipitate. The pH sensitive hydrogel (52b) showed promise for biological applications by efficiently entrapping and releasing vitamin B_12_ and cytochrome c in a pH controlled manner with retention of structural and functional efficacy.

Besides non-covalent interactions, dynamic covalent bonds have also been utilised for creating responsive soft materials and the dynamic covalent chemistry of pH responsive imine bonds inspired Yang et al. to design Schiff base-linked drug-peptide conjugate hydrogels as effective drug delivery platforms (Ren et al., 2014). They designed 4-formylbenzoic acid (FBA) capped short peptides FBA–GFFYGRGD (53) and FBA–GFFYGRGE (54) which solitarily lack gelation propensity at physiological pH (PBS, pH = 7.4) but in the presence of an amine containing anticancer drug, Gemcitabine, readily undergo Schiff base formation and form hydrogels as a result of increased hydrophobicity of the conjugate. The hydrogels displayed excellent pH triggered cancer inhibitory activities on pancreatic cancer cells and the imine linkage in both the conjugates did not attenuate the efficacy of the drug. The cancer targeting RGD sequence however endowed the RGD containing conjugate with better anticancer potency as compared to the RGE containing conjugate. Despite the highly acid labile nature of the imine linkage, the hydrogels intriguingly displayed a sustained drug release pattern under acidic environment (pH = 5) which may be attributed to the shielding of the imine linkages in the hydrophobic domains of the dense nanofibrillar network. The hydrogels act as drug reserves which release the drug in a controlled manner and the design principle portrayed here can be extended further for programmed delivery of other amine containing anticancer drugs like doxorubicin and vancomycin.

### Redox Responsive

**Scheme 2:**
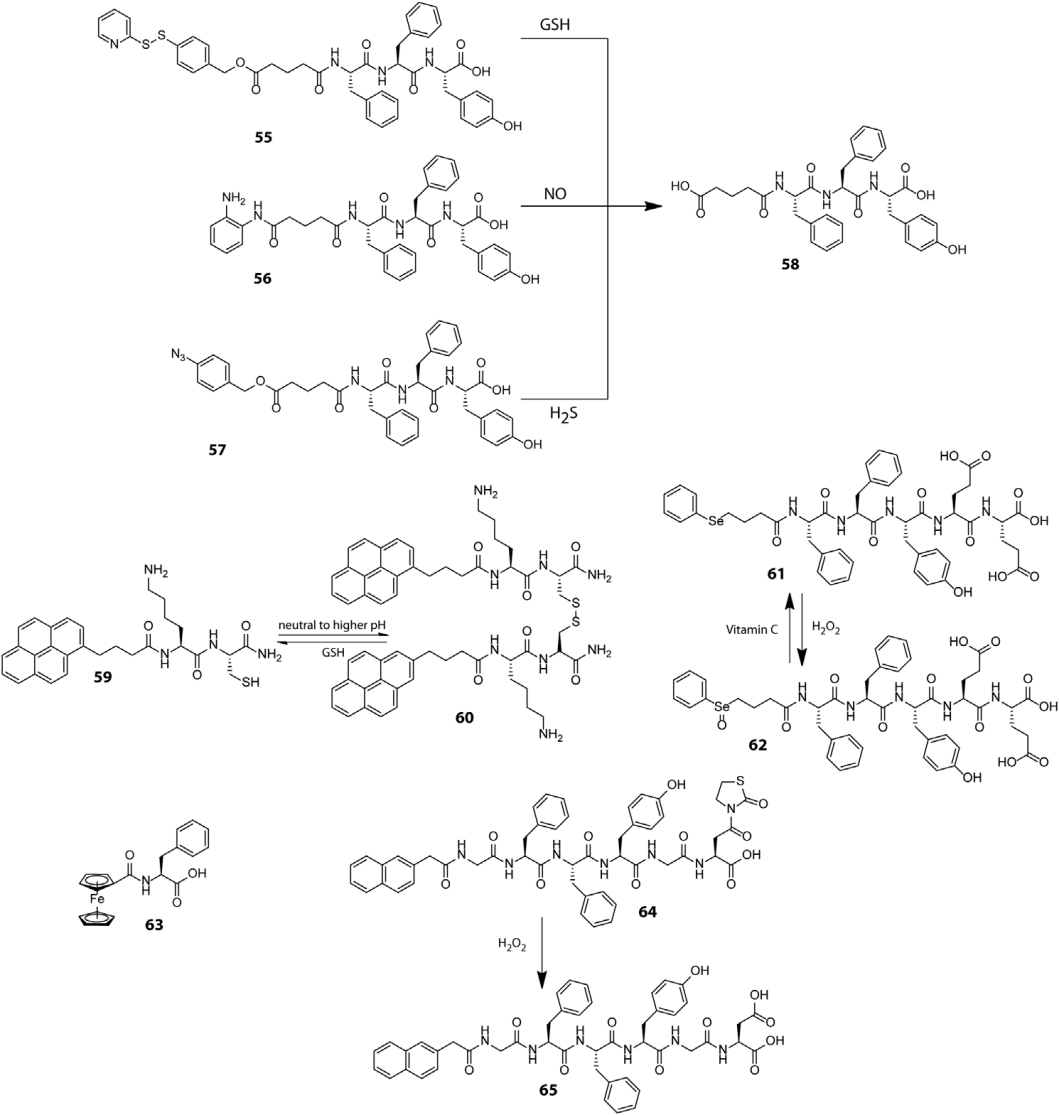
Chemical structures of representative peptides used for preparing redox responsive hydrogels with therapeutic applications.

Preserving an optimal intracellular redox balance is vital for proper functioning of critical physiological processes such as cell metabolism, proliferation and differentiation. This balance is maintained in normal cells by intricate mechanisms which regulate the levels of reactive oxygen species (ROS) and antioxidants which scavenge the ROS (Cairns et al., 2011). Rapidly proliferating cancer cells however upset this balance by upregulating ROS generation on account of their increased metabolic rate and hypoxic environment. ROS may cause irreversible damage to cells and to counteract the pernicious effects of ROS, cancer cells produce elevated levels of an antioxidant, reduced glutathione (GSH). The GSH content in cancer cells is at least 4 times as compared to normal cells and thus it acts as an important cancer biomarker (Fu et al., 2018). GSH is a well-known disulphide breaker also and this property has been extensively exploited for tailoring a wide array of tissue specific and redox responsive peptide based therapeutic soft materials. Yang et al. adopted a self-immolative approach to introduce redox responsiveness in peptide hydrogels and designed a GSH responsive self-immolative group (PBMA) capped peptide gelator PBMA-GA-FFY (55, Scheme 2) which could form hydrogel in PBS at physiological pH (Zheng et al., 2018). In the presence of GSH, the PBMA group underwent a thioquinone methide cascade reaction wherein the cleavage of the disulphide bond triggered a 1,6-elimination self-immolative step and destabilized the gelator molecule leading to the dissolution of the hydrogel. The hydrogel showed effective encapsulation and GSH triggered sustained and tuneable release of a model drug, Congo red. Easy incorporation of various stimuli responsive self-immolative groups in the progelator molecules (56 and 57) and the non-cytotoxic nature of the resulting hydrogel at low concentrations substantiated the potential of the hydrogel for drug delivery applications.

Endeavoured to develop smart responsive materials, our group using a minimalistic design principle prepared a tripeptide gelator, PyKC (59) which formed a robust and water insoluble hydrogel, a property which is atypical of low molecular weight peptide hydrogels (Singha et al., 2019c). In aqueous medium, the PyKC molecules through *in situ* disulphide formation initially form dimers (60), which further self-assemble in a stepwise manner to form tightly packed fibrillar networks of the hydrogel. The pyrene units in the gelator are lined along the outer surface of the fibrils as a result of which the hydrogel mesh acts as a hydrophobic barrier and restricts the to and fro movement of solute as well as solvent molecules from the hydrogel. This not only precludes the dissolution of the hydrogel but also bestows the hydrogel with unique compartmentalization properties which is apparent from its ability to efficiently trap and preserve the activity of enzymes even under extreme conditions of pH, heat and denaturing agents such as urea and methanol over prolonged intervals of time. The hydrogel network however degrades in the presence of disulphide cleaving agents such as TCEP and GSH, and the enzymes in their fully functional form can be easily retrieved. The ability to restrain dissolution in bulk aqueous media and withstand extreme environmental conditions together with tissue specific redox responsiveness renders PyKC an utmost contender for future biomedical applications like redox responsive drug and protein delivery vehicle.

Besides GSH, another redox active agent ubiquitously found in biological systems is H_2_O_2_ which within tolerable limits acts as an intracellular signalling agent but at heightened levels can lead to high oxidative stress and cellular damage (Ren et al., 2017). Escalated production of H_2_O_2_ in certain pathological conditions such as cancer and cardiovascular diseases has provided impetus for developing H_2_O_2_ sensitive smart drug carriers. The H_2_O_2_ induced oxidative conversion of hydrophobic dialkyl selenide group into hydrophilic selenoxide group has been widely used for controlling self-assembly properties of polymeric materials and encouraged Yang et al. to construct a redox responsive selenide-containing peptide hydrogelator (61) which assembles into a three dimensional nanofibrillar network at physiological pH (Miao et al., 2013). The oxidation of selenide to selenoxide (62) by H_2_O_2_ markedly influenced the amphiphilicity of the peptide and led to morphogenetic breakdown of the nanofibers into micelles. The micelles can be reverted back to fibrillar form by reducing the selenoxide groups using vitamin C. The interconvertible nature of the selenide group in response to biocompatible redox triggers makes this hydrogel extremely suitable for encapsulation and delivery of therapeutic agents.

Much alike the selenide group, ferrocene displays a reversible toggling of oxidation states in response to redox triggers and has been used on many occasions for designing smart materials with switchable functionalities. Zhang et al. serendipitously discovered an exceptionally simple ferrocene based peptide gelator, ferrocenoyl phenylalanine (Fc-F, 63), which self-assembled into fibrils through a stepwise hierarchical self-assembly mechanism and displayed excellent redox responsiveness (Sun et al., 2013). The aromatic interactions between the ferrocene and phenylalanine moieties drive the formation of the hydrogel and upon oxidation of ferrocene to ferrocenium ion, the aromaticity is lost and the hydrogel collapses. The reversible switching of the aromatic character of ferrocene in response to redox triggers has been utilized here for realizing reversible sol-gel transitions. The hydrogel also displayed sharp phase changes in response to pH changes and presence of host molecules. Though the therapeutic efficacy of the Fc-F hydrogel was not evaluated, the oxidative susceptibility of ferrocene in presence of H_2_O_2_ necessitates the development of ferrocene based intelligent responsive materials for therapeutic applications.

In an alternative approach, the H_2_O_2_ induced unmasking of thiazolidinone protected carboxylic acids was employed by Yang et al. to fabricate a thiazolidinone (Thi) appended peptide hydrogelator (64) wherein the oxidative removal of the thiazolidinone group (to form 65) by H_2_O_2_ disturbed the HLB of the peptide gelator and triggered a gel-sol transition (Ren et al., 2017). The hydrogel could effectively envelope the anticancer drug, gemcitabine and release it in a persistent and controllable manner in response to varying concentrations of H_2_O_2_. Further, the esterase resistant nature of the hydrogelator helped in evading inadvertent non-specific drug leakage in normal tissues.

### Enzyme Responsive

**Scheme 3:**
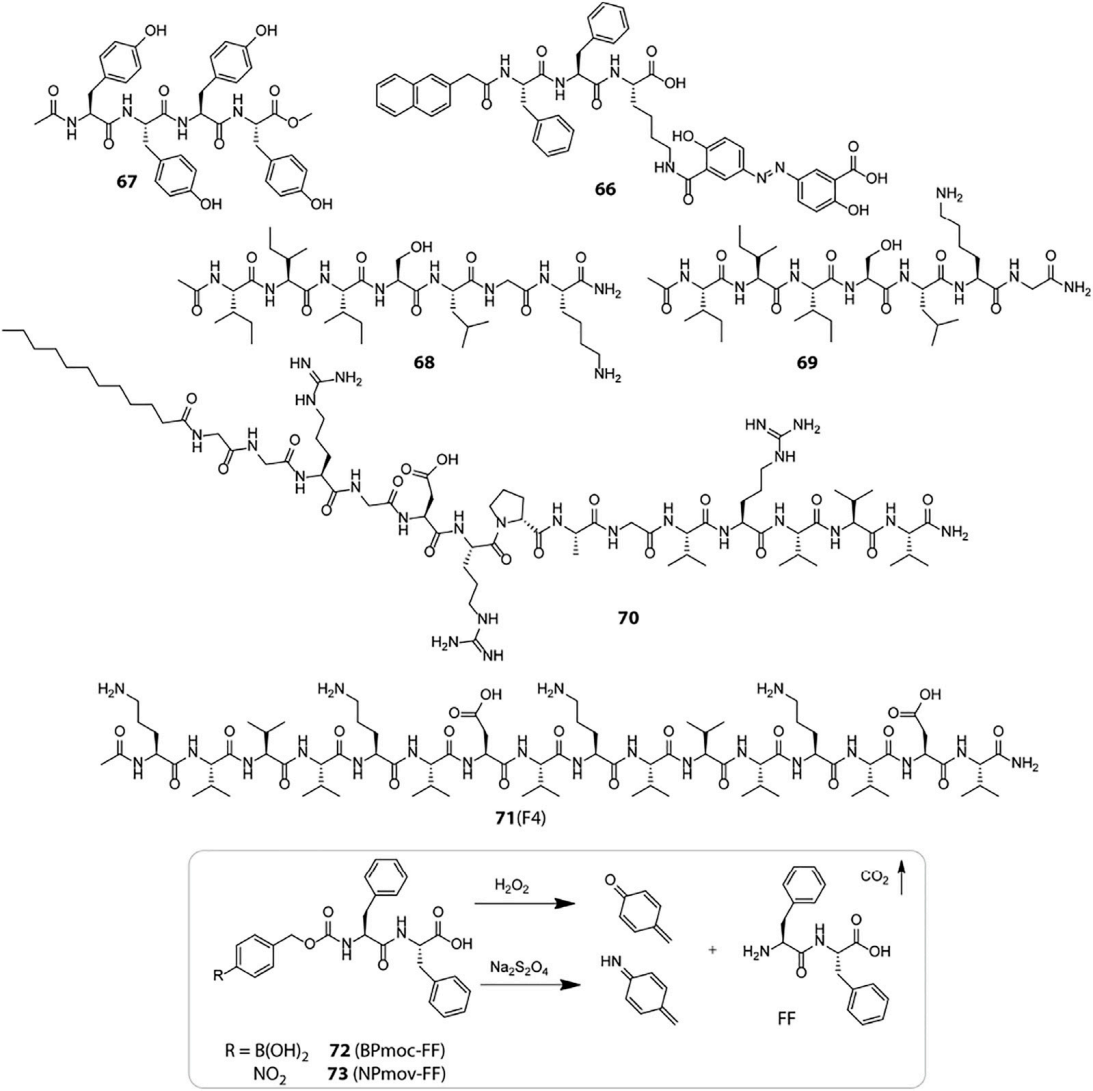
Chemical structures of representative bio-responsive peptide based smart hydrogelators used for therapeutic applications.

Enzymes are typically proteins which are characterized by their highly specific catalytic role in cellular reactions. Enzymes invariably participate in all metabolic pathways and aid critical biological processes which are vital for the sustenance of life. The high substrate specificity and overexpression of enzymes in certain specific tissues render them as effective bio-stimulants for devising highly precise and tissue specific drug delivery vehicles (He et al., 2020). The colon specific overexpression of azo reductase encouraged Xu et al. to design a peptide hydrogelator (66, Scheme 3) where the anti-inflammatory prodrug, olsalazine is directly attached to the peptide backbone (Li et al., 2010). The peptide-prodrug conjugate self-assembles in a mildly acidic solution to form hydrogel rich in β-sheet structural motifs. Azo reductase catalysed reduction of the azo linkage not only triggers a gel-sol transition but also liberates the active drug, mesalazine (or 5-aminosalicylic acid) at the site of inflammation. Unification of the prodrug in the gelator backbone proved to be an effective approach to enhancing the drug content of the hydrogel and helped in eluding non-specific drug leakage. Also, the proteolytic stability of the D-peptide variant of the hydrogel significantly enhanced its longevity in biological settings and widened the applicability of the hydrogel for wrapping additional drug molecules in the fibrillar network for combination therapy.

Enzyme triggered hydrogel degradation has been most extensively exploited for cancer treatment as the tumour tissues express unconventionally high levels of certain specific proteolytic enzymes in the TME. These enzymes not only assist in selective drug delivery but also act as hallmarks for specific cancer types. Tyrosinase is one such enzyme which is upregulated during tumorigenesis of malignant melanoma and specifically catalyses the oxidation of tyrosine. To benefit from the catalytic activity of tyrosinase, Yang et al. designed a tyrosine rich peptide gelator, Ac-YYYY-OMe (67) which forms hydrogel at physiological pH but undergoes dissolution in response to tyrosinase enzyme (Gao et al., 2011). The π−π stacking interactions between the aromatic tyrosine residues is mainly responsible for holding up the hydrogel network together. The catalytic conversion of tyrosine to quinone results in the loss of aromatic π−π interactions and consequent hydrogel degradation. Owing to the enzyme responsive and innocuous nature of the gelator, the tyrosinase triggered gel-sol transition of the hydrogel was evaluated for *in vitro* release of a model drug, congo red. A sustained release pattern was observed only in the presence of tyrosinase which highlights the potential of the hydrogel for long term cancer medication.

Minute variations in peptide sequences can have huge implications on enzyme responsiveness of peptide hydrogels, which was exemplified by Xu et al. through their report on MMP-2 sensitive peptide hydrogels (Chen et al., 2017). MMP-2 is a class of matrix metalloproteinase upregulated in cancer cells and they have a strong affinity for serine at P1 and leucine at P1′, respectively (The cleavage site includes residues both N- and C-terminal to the scissile bond, denoted as …P3-P2-P1-P1′-P2′-P3′… when the cleavage occurs between P1 and P1’ residues). An enhancement in site specificity for the S-L bond is observed when the N-terminal side contains hydrophobic residues (e.g., Iso and Val), and the C-terminal side contains basic amino acids (e.g., Arg and Lys) at P2′ and small residues (e.g., Gly and Ala) at P3’, respectively. Based on these design principles, two peptide gelators, Ac-I_3_SLKG-NH_2_ (68) and Ac-I_3_SLGK-NH_2_ (69), were designed out of which only the former is cleaved by MMP-2 into Ac-I_3_S and LKG-NH_2_ whereas the latter is not. The MMP-2 sensitive hydrogel of 68 exhibiting β-sheet conformation was used for entrapping a FITC labelled anticancer peptide G (IIKK)_3_I-NH_2_ (FITC-G3) and incubated with HeLa cells which are known to overexpress MMP-2. The enzymatic degradation of the hydrogel network gradually lowered the mechanical strength of the hydrogel and released the anticancer peptide in a “cell-demanded” fashion, which markedly inhibited cancer cell proliferation.

Merely engineering self-assembling peptides with enzymatic substrates may not always suffice for effective enzyme responsiveness in hydrogels as in the aggregated state, the peptide substrates are mostly buried in the tightly packed fibrils and hardly accessible to the macromolecular enzymes. To enhance the enzyme-substrate interaction and thereby the responsiveness of enzyme sensitive peptide hydrogels, Cui et al. presented a reverse bolaamphiphile (RBA) design which exposes the MMP-2 cleavable groups at the surface of the peptide filaments (Chakroun et al., 2020). The RBA-1 gelator (70) consists of two lateral hydrophobic domains: a dodecyl group at the N-terminus and high β-sheet propensity triple valine residues at the C-terminus; and a central hydrophilic stretch: the cell adhesive RGDR sequence and MMP2 labile PLGVR segment. RBA-1 hydrogel displayed extensive filamentous network with the hydrophilic scissile bonds exposed at the filament surface. MMP2 treatment cleaved off the VRVVV segment and disrupted the hydrogel to micellar aggregates. To emphasize the importance of the RBA design, they designed a control molecule (C12-GGPLGVRVVVRGDR) where the hydrophilic RGDR sequence is shifted to the C-terminus and the VVV sequence is pushed towards the N-terminus. The target substrate (PLGVR) was now obscured in the filamentous network and the hydrogel resisted MMP2 triggered degradation over a prolonged duration of 2 weeks. Another control molecule with a scrambled PLGVR sequence also resisted MMP2 degradation which substantiated that the accessibility and specificity of the target substrate is key to achieving enzyme mediated disassembly. Finally, the efficacy of the RBA design for drug delivery was validated by designing a paclitaxel (PTX) bearing RBA gelator (DRBA-1) which self-assembled into a hydrogel similar to RBA-1 and disintegrated in the presence of MMP-2 to liberate the active drug in a controlled manner. The unique RBA design showed great potential for amplifying enzyme sensitivity of peptide hydrogels and can be further explored for developing various enzyme responsive drug depots that dispense therapeutics in a “cell-controlled” fashion.

Exogenously trapped enzymes have also been used to elicit enzyme responsiveness in peptide hydrogels in response to certain specific biomolecules present in the biological systems. Glucose Oxidase (GOx) is one such enzyme that has been widely employed to induce glucose responsiveness in a variety of self-assembled delivery systems. Glucose, the prime source of energy in all living organisms, is metabolised and stored in the body as glycogen by a pancreatic hormone, insulin which is secreted in response to elevated levels of blood glucose. An impaired production and/or functioning of insulin causes an increase in blood glucose level (hyperglycaemia) and leads to a metabolic disorder, Diabetes mellitus. The primary treatment available for diabetes at present is exogenous insulin injections which besides being painful, requires regular blood glucose monitoring to avoid over dosage and development of hypoglycaemia. A better and safer approach is to use insulin embedded hydrogels which regulate insulin delivery in a controlled manner in response to high glucose levels. Qian and Ge et al. installed a glucose fuelled enzymatic acidification reaction within pH sensitive peptide hydrogels to realise consistent insulin delivery *in vitro* and *in vivo* (Li et al., 2017). They employed a bi-enzymatic bio-catalytic reaction network wherein glucose oxidase (GOx) converts glucose to gluconic acid and reduces the local pH; and catalase (CAT) breaks down the by-product of GOx oxidation, H_2_O_2_ into water and oxygen and promotes the catalysis of GOx. They designed a modified version of the MAX1 peptide, IA-2 (17, Figure 2) with better pH responsiveness and immobilised the enzymes along with FITC-insulin in the hydrogel network to accomplish glucose responsive sustained insulin release. Later, they redesigned another pH sensitive peptide gelator, RATEA16 by substituting Ala with Val and Iso residues, and Arg with Orn residues to create a library of peptide gelators with superior stability and pH sensitivity. Utilizing the same bi-enzymatic reaction network, the most viable peptide hydrogel, F-4 (71) displayed efficient regulation of blood glucose levels in biological models for up to 8 days (Fu et al., 2018). The enzyme mediated localized acidification reaction presents an easy yet effective strategy that can be extended to a variety of pH responsive drug delivery platforms.

The H_2_O_2_ generated during glucose oxidation can be used for inciting glucose responsiveness in redox active systems as well. Hamachi et al. presented two redox responsive hydrogelators, BPmoc-FF (72) and NPmoc-FF (73) which get solvated in response to oxidative and reductive environments, respectively (Ikeda et al., 2011). The H_2_O_2_ sensitive BPmoc-FF was assessed for its drug delivery proficiency using FITC labelled insulin and to fine tune the rate of hydrogel dissolution and thereby the rate of drug release, glucose oxidase (GOx) was immobilized in the hydrogel network to produce H_2_O_2_ *in situ* from glucose in a highly precise and controlled fashion. The glucose triggered amplification of H_2_O_2_ production and hydrogel degradation displayed a sustained release kinetics for FITC-Insulin which can be highly beneficial for targeted drug delivery in diabetic patients.

## Bio-Sensitized Intracellular Hydrogelation

The advancement in supramolecular chemistry over the decades has led to a deeper understanding and insight into the structure-property relationship of peptide building blocks and has empowered the development of therapeutic peptide based hydrogels with tuneable mechanical properties, porosity, functionalities and bio-responsiveness. Meticulously designed peptide hydrogel based drug delivery systems have helped in surmounting major pharmacodynamic predicaments such as low solubility and stability of drugs, poor bioavailability, non-specificity and systematic toxicity (Webber and Pashuck, 2021). However, to harness the full potential of peptide hydrogels for biomedical applications, developing practically feasible strategies to implant hydrogels at the ailing tissue sites becomes highly imperative. Surgical implantations, besides being risky and convoluted, may lead to various post-surgical complications, thereby further adding to the patient’s suffering. Precise implantation of drug loaded hydrogels at deep and intricate tissue locations without any side effects still remains an extreme medical challenge. Injectable hydrogels present a non-invasive alternative and significantly increase the patient compliance but its applicability is mostly limited to subcutaneous tissues only (Sis and Webber, 2019). So instead of implanting exogenously formed hydrogels within tissues, a much appealing approach is to generate the hydrogel nanofibers within the cells itself. A well explored strategy is to design stimuli responsive progelator molecules which remain in monomeric or nano-dimensional aggregated form under physiological conditions but upon encountering certain specific stimulants lead to *in situ* generation of active gelators that can readily form hydrogels. The aqueous progelator solution can be easily injected intravenously into the blood stream and transported to the target site through the circulatory pathway. Ligation of suitable targeting groups may help in directing the progelators to specific tissues where they can be readily absorbed, accumulated and subsequently converted to active gelators that can self-assemble into entwined fibrillar networks within the cells. Functionalization of the progelator molecules with suitable therapeutics that do not alter the self-assembly process may be a step further to *in situ* generation of hydrogel drug depots within the diseased cells for long term medication with minimum systematic toxicity.

Designing stimuli responsive progelator molecules generally, but not exclusively, follow one of the following three strategies:

1) Incorporation of stimuli responsive functionalities (e.g., pH responsive amino acids) in the progelators that can respond to specific physicochemical environment of the target tissues and trigger conformational switching to generate the active gelators. Such progelators involve stimuli triggered reprogramming of non-covalent interactions only and do not involve any covalent modifications.
2) Engineering structural constraints in gelator molecules that impede their gelation ability. Stimuli triggered relaxation of the structural constraints reactivate the gelators towards gelation. Such progelators invariably involve stimuli triggered bond cleavage as the activation step.
3) Designing small peptide fragments that intrinsically lack gelation propensity but upon stimuli triggered cross-linking lead to formation of extended networks of hydrogel nanofibers. Such progelators require a covalent/non-covalent bond formation step to generate the active gelator.

Based on the above strategies, several chemically as well as enzymatically triggered self-assembling systems have been documented which are discussed in the subsequent sections.

### Chemically Induced *in situ* Hydrogelation

The disparity in physicochemical parameters across different cells and tissues serve as biological signals that can trigger transformation of peptidic progelator molecules into active gelators and elicit structural evolution of the resulting peptide building blocks into well knitted nanofibrillar networks within cells. Also, the aqueous cellular compartments mediate a multitude of biochemical reactions which can be emulated and applied to synthetic analogues for *in situ* generation and assembly of gelator molecules. Nilsson et al. developed a redox triggered self-assembling peptide by cyclizing a β-sheet forming sequence Ac-C(FKFE)_2_CG-NH_2_ (74, Table 2) via disulphide bonding of the fringing cysteine units (Bowerman and Nilsson, 2010). The conformationally locked macrocyclic peptide lacks gelation propensity and remains solvated in aqueous medium. Reductive linearization of the cyclic peptide by TCEP or DTT, however revoked the conformational constraint and led to the formation of a rigid, viscoelastic hydrogel encompassing amyloid like fibrillar superstructures. Apart from reductive triggers, various other stimuli responsive chemical bonds can be employed for acquainting conformationally restrained cyclic progelator peptides with environmental responsiveness and thus the design strategy depicted here can be easily customized for selectively triggering *in situ* hydrogelation in diverse cells and tissue types.

**TABLE 2:** Examples of bio-sensitized peptide hydrogels.

Stimuli	Peptide sequence (short name)	Hydrogel degradation mechanism	Ref
Redox	Ac-C(FKFE)_2_CG-NH_2_ (74)	TCEP/DTT triggered linearization of conformationally constrained macrocyclic peptide leads to hydrogelation	Bowerman and Nilsson (2010)
pH	BP-KLVFF-His_6_-PEG (75)	HLB modulation owing to protonation of the His residues at acidic pH triggers hydrogelation	Yang et al. (2017)
Metal ion	BP-KLVFF-RGD (BKR, 76)	Metal-ion coordination between Ca^2+^ ions and Aspartate residues of the peptide progelators triggers nanoparticles to nanofibers transition	Xu et al. (2016)
Carboxylesterase (CES)	NapFF-Tau (Tau: Taurine, 77)	CES catalysed abstraction of the taurine groups triggers nanofibrillation	Li et al. (2015)
Alkaline phosphotase (ALP)	NapFFKYp (78)	Dephosphorylation of the tyrosine phosphorous ester triggers micelle to nanofibers transformation	Huang et al. (2015)
MMP-9	GFFLGLDD, PhAc-FFAGLDD (79)	Enzymatic cleavage of hydrophilic DD segment triggers micelle to nanofibers transition	Kalafatovic et al. (2016)
Transglutaminase (TGase)	I_3_QGK (80)	Enzyme mediated peptide dimerization via intermolecular ε-(γ-glutamyl)lysine isopeptide bonding triggers nanofibrillation	Chen et al. (2016)
Enterokinase (ENTK)	Nap-^D^F^D^F^D^K (^ε^G-FLAG)-NBD (81)(FLAG: DYKDDDDK, NBD: Nitrobenzoxadiazole)	Enzymatic proteolysis of the hydrophilic FLAG tag induced micelle to nanofiber evolution	He et al. (2018)

**Scheme 4:**
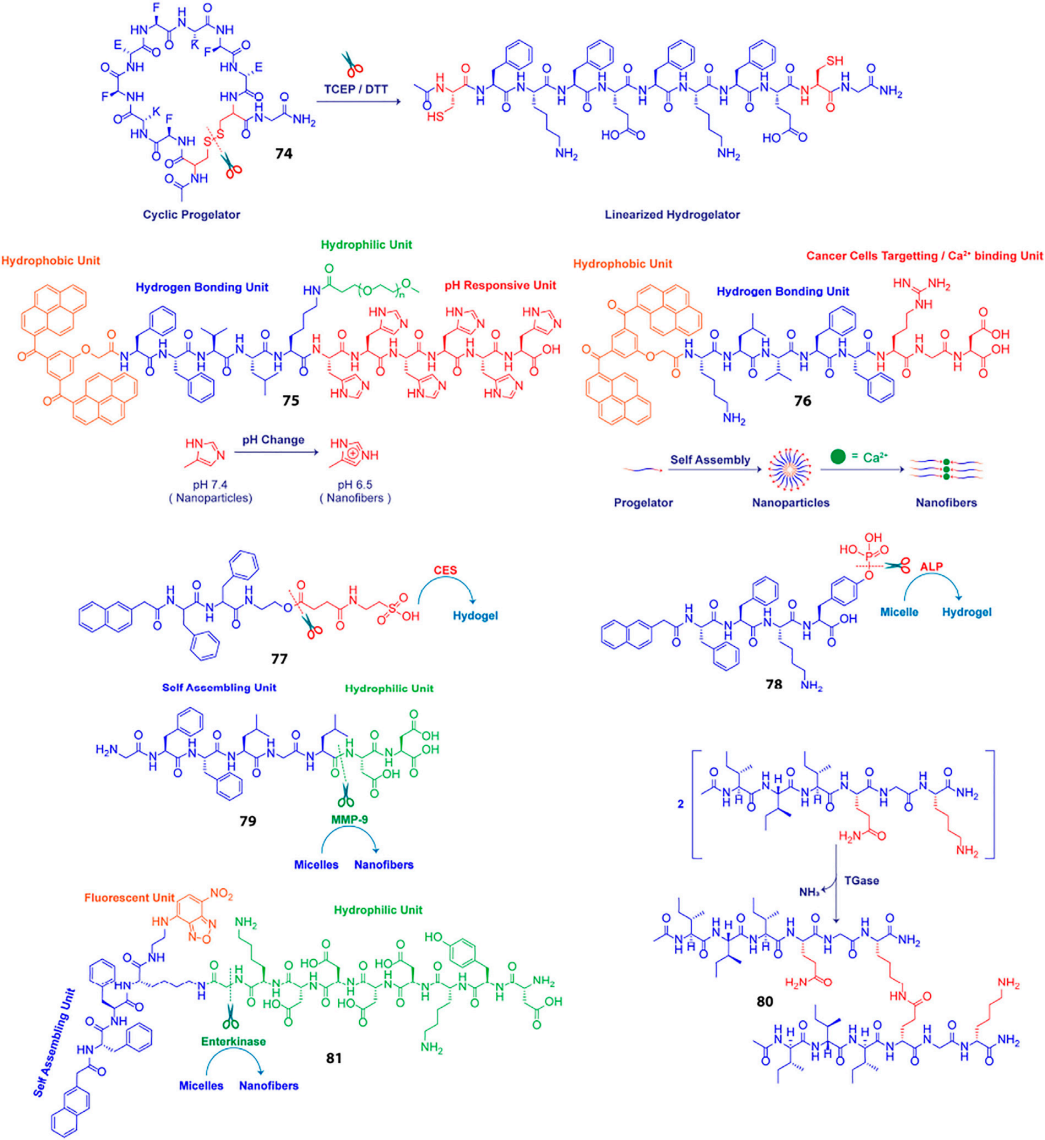
Schematic presentation of different chemically and enzymatically induced *in-situ* hydrogelation processes.

Wang et al. employed an alternative strategy to enrich tumour tissues with therapeutic agents. They constructed a Y-shaped peptide, 75 (Scheme 4), comprising a β-sheet forming KLVFF sequence flanked by laterally positioned hydrophobic bis-pyrene and hydrophilic PEG units at the N and C termini, respectively and a pH switchable His_6_ segment functionalized to the lysine side arm (Yang et al., 2017). The peptide initially aggregates into nanoparticles at physiological pH (7.4) and can be easily injected and accumulated at tumour sites via a passive targeting mechanism. Protonation of the His residues at the intratumoral pH (6.4) altered the HLB of the peptides and induced morphological transformation of the nanoparticles into nest-like β-sheet nanofibrillar networks. Intravenously injected hydrophobic guest molecules introduced post nanofibrillation were intercalated and trapped in the hydrophobic domains of the nanofibers and retained in the confined tumoral space for up to 96 h. The hydrophobic fluorophore, Nile red (NR) upon getting locked in the β-sheet domains of the nanofibers displayed 7.5-fold higher fluorescence intensity as compared to free NR treated tissues after 4 days of injection. Meanwhile, the hydrophobic therapeutics, indocyanine green (ICG) and doxorubicin (DOX) were also hosted and enriched in tumour tissues post injection and displayed highly potent photothermal and chemotherapeutic effects, respectively over prolonged durations. The stimuli responsive *in situ* formation of nanofibrillar hosts presents a generic approach to homing theranostics in target tissues and may be particularly helpful in cases where direct pairing of drugs with the gelator backbone is not feasible.

Impelled by the fact that different types of metal ions are profusely distributed in the biological system and facilitate self-assembly of a variety of proteins into their native forms, Wang et al. using similar structural elements as discussed above, designed an amphiphilic supramolecular peptide module, BP-KLVFF-RGD (BKR, 76) which self assembles into fluorescent nanoparticles in aqueous medium and transforms into β-sheet nanofibers in the presence of Ca^2+^ ions(Xu et al., 2016). Owing to the ability of the aspartate residue of RGD sequence to coordinate with Ca^2+^ ions at the “metal ion-dependent adhesion sites” (MIDAS) of integrin α_v_β_3_ transmembrane proteins overexpressed on many cancer cell types, the BKR nanoparticles with surface-exposed RGD motifs preferentially adhered to U87 cancer cells with overexpressed α_v_β_3_ integrin and underwent *in situ* morphological transformation to nanofibers on the cell surface. Surface nanofibrillation aided cancer cell imaging by specifically and selectively lighting up the cancer cells surfaces with characteristic green fluorescence emerging from the aggregated bis-pyrene units in the nanofibers. Moreover, the enwrapment of the U87 cells in the nanofiber network blocked their communication with the surrounding extracellular matrix (ECM) and led to anoikis. Stimuli triggered structural evolution of peptide-based superstructures on infected cell surfaces may thus be exploited as an effective “drug-free” therapeutic strategy to cripple essential metabolic activities of diseased cells, eventually leading to apoptosis in a highly precise and controlled fashion.

### Enzyme Induced *in situ* Hydrogelation

A key strategy to realize enzyme instructed self-assembly is to engineer known self-assembling peptide building blocks with enzyme cleavable hydrophilic moieties that impede the gelation of the resulting progelators under physiological settings but makes them susceptible to intracellular nanofibrillation upon encountering the targeted enzyme in the disease related cells and tissues. Dinulescu and Xu et al. exemplified this approach by designing small peptide precursor (77) wherein the self-assembling peptide motif Nap-FF is ligated to hydrophilic taurine molecule through a carboxylesterase (CES) susceptible ester linkage (Li et al., 2015). Co-administration of the gelator precursor along with cisplatin in ovarian cancer cells resulted in CES catalysed abstraction of the taurine groups, leading to intracellular formation of nanofibrils and drastically enhanced the anticancer potency of cisplatin.

Chen et al. presented a notable example of morphology transition where phosphatase triggered transformation of indocyanine green (ICG) loaded peptide micelles to spatiotemporally localized ICG doped co-assembled nanofibers was realized for targeted photothermal therapy (Huang et al., 2015). They fabricated an alkaline phosphatase (ALP) responsive peptide progelator NapFFKYp (78) which formed micelles in aqueous medium and could effectively trap amphiphilic ICG molecules in the micellar assembly. Dephosphorylation of the tyrosine phosphorous ester at the ALP overexpressing tumour tissues led to conversion of micellar nanoparticles into persistent β-sheet nanofibers with the ICG molecules accommodated in the fibrillar domains. ICG molecules were doped in the fibres as J-aggregates which induced a red-shift and hence, markedly enhanced the NIR absorption of ICG. This in turn boosted the photothermal conversion efficiency of the locally enriched photoacoustic ICG molecules at the tumour sites and enabled dual modality imaging guided photothermal therapy. Further, *in vivo* studies revealed that the aggregation induced retention of ICG provided a long imaging time window of 24–48 h and complete eradication of tumour cells was achieved through high precision laser irradiation.

Likewise, Ulijn et al. demonstrated transformation of drug laden micellar nanocarriers to nanofibrillar drug depots employing a MMP-9 responsive peptide GFFLGLDD (79) (Kalafatovic et al., 2016). Similar to their previously reported MMP-9 sensitive peptide PhAc-FFAGLDD, this peptide could also assemble into micelles in aqueous medium with doxorubicin (DOX) embedded in the hydrophobic core. MMP-9 digestion of the micelles in tumour tissues severed the hydrophilic DD segment from the amphiphilic peptide yielding the active hydrogelator GFFLGL along with small amounts of residual peptide fragments such as GFF and GF. The β-sheet forming GFFLGL peptide generated fibrillar nanostructures at the tumour tissues and accumulated DOX in the hydrophobic pockets of the nanofibrous peptide depot. Slow decomposition of the biodegradable peptide filaments gradually released the trapped DOX for enhanced and localized chemotherapy over extended periods of time. Enzymatic cleavage directed autonomous transition of peptide nanovectors to fibrillar drug repositories thus presents an attractive approach to augment the effectiveness of drugs at diseased tissue sites and minimize toxicity to surrounding tissues and organs.

Unlike typical enzymatic scission directed self-assembly processes, Lu and Xu et al. reported an enzymatic dimerization mediated hydrogelating system (Chen et al., 2016). They designed an amphiphilic short peptide I_3_QGK (80) which despite forming elongated nanoribbons in aqueous solution remains solvated and displays distinct sol-gel transition in the presence of transglutaminase (TGase). The peptide essentially exists as a mixture of assembled nanoribbons and non-assembled monomers in aqueous solution. As the enzyme sensitive Q residues are concealed in the hydrophobic regions of the nanoribbons, only the free monomers participated in the TGase mediated intermolecular ε-(γ-glutamyl)lysine isopeptide bonding and formed lipid like dimers with two hydrophobic peptide chains and a single charged amino acid head group. The dimers with higher β-sheet propensity assembled into fibres which in conjugation with the pre-existent nanoribbons formed densely entangled nanofibrillar hydrogel. In biological systems, TGase (Factor XIIIa) is generated *in situ* via activation of Factor XIII by thrombin during bleeding. The *in situ* generated TGase enabled rapid coagulation of I_3_QGK treated blood at the injured site and dramatically reduced blood loss in animal models. Moreover, I_3_QGK also stimulated platelet adhesion at the site of injury and induced rapid and effective homeostasis. Also, the peptide presented negligible haemolytic response and low cytotoxicity against normal mammalian cells which is extremely desirable for real life clinical applications.

Enzyme instructed self-assembly (EISA) of peptidic drug vectors has most extensively been employed for immobilising drugs in and around the ailing cells and tissues. The actual sites where the drug molecules exert their therapeutic actions are however the subcellular organelles and thus EISA mediated retention of drugs at the organelles is a highly sought after strategy to improve overall drug potency and specificity. In this direction, Xu et al. designed branched peptide progelators which afforded micelles capable of delivering DOX as well as proteins into cells, specifically to mitochondria (He et al., 2018). The branched peptide, Nap- ^D^F^D^F^D^K (^ε^G-FLAG)-NBD (81) comprised of a hydrophilic enterokinase (ENTK) susceptible and mitochondria targeting FLAG-tag (DYKDDDDK) attached via a glycine spacer to the lysine residue of a self-assembling peptide, Nap-FFK and a nitrobenzoxadiazolyl ethylenediamino moiety (NBD-EA) at the C-terminus as the fluorescence reporter. Electrostatic interactions between the negatively charged FLAG-tag moieties on the corona of the micelles and the positively charge intermembrane space of mitochondria directed the micelles towards mitochondria where proteolysis of the hydrophilic peptide branch by intracellular ENTK transformed the micelles into nanofibers. Mitochondrial localization of the nanofibers was corroborated by the enhanced fluorescence of NBD probe in the fibrillar matrix. Fluorescence imaging also revealed that the DOX loaded FLAG-tagged micelles enhanced DOX uptake in mitochondria and synergistically inhibited cancer proliferation by damaging mitochondrial DNA. The FLAG-tag moiety unlike most lipophilic and cationic mitochondria targeting groups does not induce any cytotoxicity upon accumulation and thus presents an elegant alternative for designing mitochondria specific drug couriers.

## Therapeutic Applications

### Therapeutic Delivery and Combination Therapy

Delivery of a therapeutic agent (drug, protein, hormone or gene) to the desired location inside the body is one of the most challenging tasks (Webber and Pashuck, 2021). It is obvious that hydrogel based systems cannot be injected intravenously. As discussed earlier, if a drug loaded hydrogel needs to be used for localized delivery, it is essential that the gel possesses injectability property (Sis and Webber, 2019). However, it is clear from literature that most of the reported systems are of composite nature where a polymer is used along with peptides/proteins to prepare injectable therapeutic hydrogels.

One of the prime goals of injectable drug delivery hydrogels is to treat cancerous growth. In this context, new approaches for cancer therapy not only require rapid killing of primary tumors but also demand systems that can control over the tumor microenvironment to deplete immunosuppressive cells and prime effector immune cells. Peptide based injectable hydrogels are suitable candidates to do so. For example, an extremely potent anticancer agent, Melittin, is conjugated with a well-studied peptide hydrogelator (RADA)_8_ to prepare a fusion peptide hydrogelator, MR (Table 3) (Jin et al., 2018) The conjugated peptide can form thixotropic hydrogel in the physiological pH range and drugs like DOX can be efficiently loaded into the gel matrix. When DOX loaded MR hydrogel is injected to B16-F10 tumor-bearing mice, the presence of melittin dramatically reduced the side effects and increase the antitumor efficacy and immune responses. Dual delivery from the hydrogel causes a sustained antitumor effect along with an increased immunity, decreased immunosuppression, and activation of effector T cells.

**TABLE 3:** Examples of peptide hydrogels used for therapeutic applications.

Sequence (short name)	Application	Ref
Melittin-(RADA)_8_ (MR)	Codelivery of melittin and DOX	Jin et al. (2018)
^D^F^D^F^D^Y	Inhibition of cancer proliferation	Kuang et al. (2014)
Nap-^D^F^D^F^D^K^D^Y(Phospho)	Inhibition of cancer proliferation	Li et al. (2013)
Nap-G^D^F^D^F^D^YGRGD	Delivery of 10-hydroxycamptothecin	Yang et al. (2015)
Fmoc-FFRGDF (82)	Delivery of 5-fluorouracil	Xu et al. (2010)
Ac-(RADA)_4_-CONH_2_	Delivery of various proteins	Koutsopoulos et al. (2009)
Ac-KLDLKLDLKLDLRR-CONH_2_ (KLD2R)	Codelivery of TNF-α and HGF	Liu et al. (2020)
K(SL)_3_RG (SL)_3_KGRGDS	Bimodal delivery of growth factors (GFs) and cytokines	Wickremasinghe et al. (2014)
MAX8	Co-delivery of erlotinib and DOX.	Majumder et al. (2018)
LIVAGKC and LK_6_C	Healing of full-thickness excision wounds in mouse	Seow et al. (2016)
Ac-ILVAGK-NH_2_ and Ac-LIVAGK-NH_2_	Burn wound healing in mouse model	Loo et al. (2014)
Nap-GFFKH	Dual release of antibiotic and FGF-2 for wound healing	Cui et al. (2020)
QHREDGS	To cure wounds in diabetic mouse	Xiao et al. (2016)
K_2_(SL)_6_K_2_	To cure wounds in diabetic mouse	Carrejo et al. (2018)
A_9_K_2_	Highly selective antibacterial activity	Bai et al. (2016)
H_2_N-(CH_2_)nCONH-Phe-CONHC_12_ (n = 1–5, C_12_ = dodecyl; [P1 (n = 1), P2 (n = 2), P3 (n = 3), P4 (n = 4), P5 (n = 5)])	Antimicrobial activity against Gram-positive bacteria (*Staphylococcus aureus, Bacillus subtilis*) and Gram-negative bacteria (*Escherichia coli*)	Nandi et al. (2017)
C_15_H_31_CONH-NAVSIQKKK-CONH_2_	Antimicrobial activity	Adak et al. (2019)
Fmoc-L-Phe + OTE-D-Phe 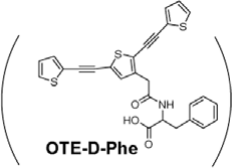	Efficient capture and specific killing of antibiotic-resistant Bacteria, *Staphylococcus aureus*	Zhao et al. (2019)
Fmoc-W, Fmoc-M, Fmoc-Y	Selectively inhibit the growth of Gram-positive bacteria	Xie et al. (2020)
Ac-(3′-PyA)LRLRLRL (3′-PyA)CONH_2_ + Ag(I); (3′-PyA = 3′-pyridyl Alanine)	Ag(I) release to kill *Escherichia coli* and *Staphylococcus aureus*	D’Souza et al. (2020)
Ac-(RADA)_4_GGSKPPGTSS-CONH_2_ (SKP)	To create a 3D cell environment for human adipose stem cells	Liu et al. (2013)
Ac-(RADA)_4_GGFHRRIKA-CONH_2_ (FHR)		
Ac-(RADA)_4_GPRGDSGYRGDS-CONH_2_ (PRGD)		
Protein G conjugate of EAK16 (G_EAK)	As 3D matrix to support thymic epithelial cells (TECs) and differentiation of various T-cell subsets *in-vivo*	Liu et al. (2019)
Ac-(RADA)_4_-CONH_2_ (PuraMatrix™)	Bone regeneration in bone defects of calvaria in mice	Misawa et al. (2006)
FEFEFKFK	Osteogenic differentiation of hMSC	Castillo Diaz et al. (2016)
C_15_H_31_CONH-CCCCGGGS(PO_4_)RGD	Biomineralization of hydroxyapetite	Hartgerink et al. (2001)
QK + RGD	Enhancement of angiogenesis and osteogenesis	Hung et al. (2020)
KVKEVFFVKEVFFVKEVY + CNT	Augmenting neural signal	Nam et al. (2020)
Ac-ILVAGK-CONH_2_	As bioink with hMSCs for 3D Bioprinting	Loo et al. (2015)
C_15_H_31_CONH-V_3_A_3_VPGIGK_3_-CONH_2_	3D Bioprinting	Hedegaard et al. (2018)
C_15_H_31_CONH-V_3_A_3_H_2_K-CONH_2_		
C_15_H_31_CONH-V_3_A_3_K_3_-CONH_2_		
C_15_H_31_CONH-V_3_A_3_E_3_-CONH_2_		
PyKC	Bone regeneration	Chowdhuri et al. (2021)

The use of drug-loaded hydrogels for localized delivery is not only aimed to reduce the severe side effects of the therapeutics but also to achieve sustained release of the drug. In this regard, peptide based systems suffer from easy dissolution through hydrolysis catalyzed by endogenous peptidases. To counter that, D-amino acid based systems is an excellent choice. Xu reported the enzyme triggered self-assembly of a D-tripeptide (^D^F^D^F^D^Y) and utilized this hydrogel to inhibit the growth of cancer cells (Kuang et al., 2014). The same group also reported the hydrogelation of another D-peptide (Nap-^D^F^D^F^D^K^D^Y(Phospho)) triggered by enzymatic dephosphorylation and utilized this hydrogel to inhibit the growth of Hela cells (Li et al., 2013). The *in-vivo* stability of D-peptide hydrogels was also shown by Yang and co-workers by using Nap-G^D^F^D^F^D^YGRGD hydrogel for delivery of 10-hydroxycamptothecin (HCPT) in mouse model (Yang et al., 2015).

**FIGURE 4:**
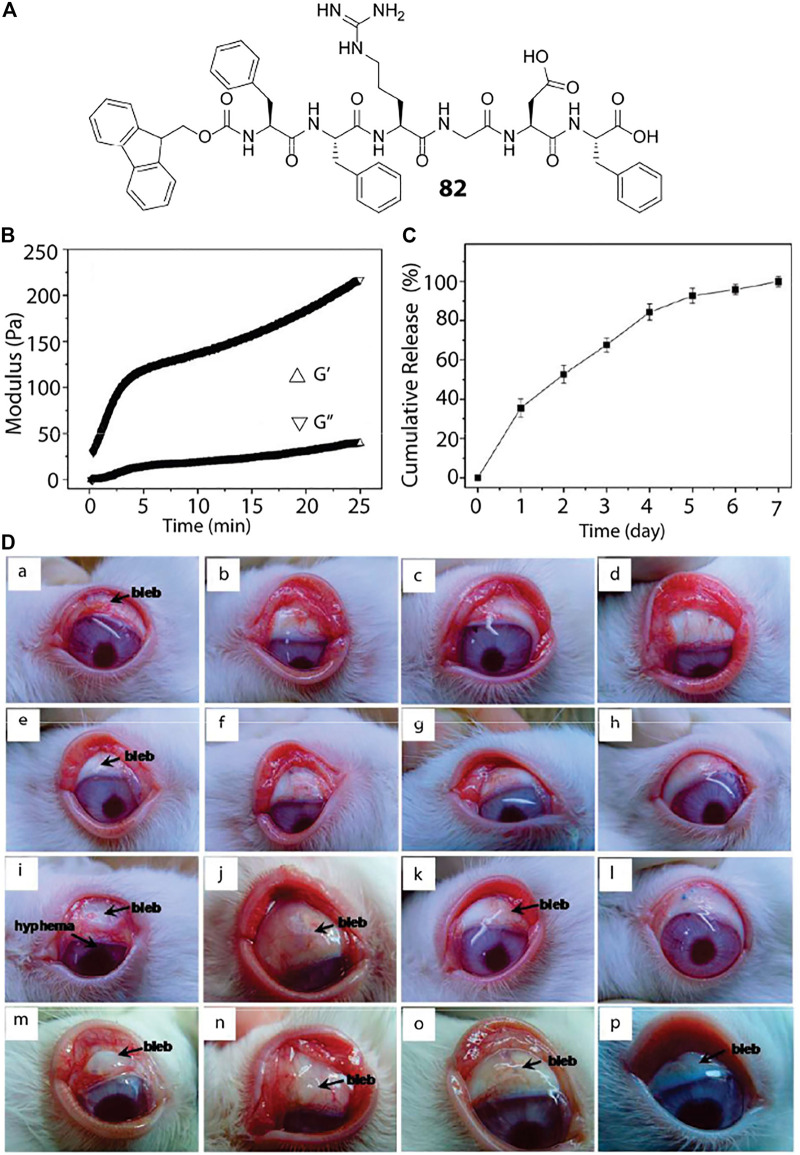
Peptide hydrogel based ocular drug delivery. **(A)** Chemical structure of the peptide hydrogelator used for ocular delivery of 5-Fu. **(B)** Oscillatory rheology of the peptide hydrogel where the storage and loss modulus are plotted against time. **(C)** *In-vitro* 5-Fu release by the hydrogel under physiological condition showing sustained release of the drug over a period of 7 days. **(D)** (a–d): Photo images of the rabbit eyes that underwent filtering surgery alone at postoperative (a) 7, (b) 14, (c) 21, and (d) 28 days, respectively; (e–h) photo images of the rabbit eyes that received the peptide hydrogel at postoperative (e) 7, (f) 14, (g) 21, and (h) 28 days, respectively; (i–l) photo images of the rabbit eyes that exposed to 5-Fu intraoperatively at postoperative (i) 7 (j) 14 (k) 21, and (l) 28 days, respectively; (m–p) photo images of the rabbit eyes that received the 5-Fu-loaded peptide hydrogel at postoperative (m) 7 (n) 14 (o) 21, and (p) 28 days, respectively. Reproduced with permission (Xu et al., 2010). Copyright 2010 American Chemical Society.

Apart from cancer treatment, hydrogels can also be used as a carrier and delivery vehicle for other drugs, proteins, genes, hormone etc. The glucose mediated insulin delivery by an injectable hydrogel is discussed in *Enzyme Responsive*. Delivery of ocular drugs have been achieved with the help of short peptide based hydrogels. A modified RGD sequence that read as Fmoc-FFRGDF (82, Figure 4) is used to prepare an injectable hydrogel loaded with anti-proliferating model drug, 5-fluorouracil (5-Fu, Figure 6) (Xu et al., 2010) Administration of this 5-Fu-loaded peptide hydrogel in the filtering surgery of rabbit eyes resulted in sustained release of 5-Fu to inhibit the scleral flap fibrosis effectively.

Delivery of proteins can also be achieved using biostable and bioresponsive peptide based hydrogels. It is important that the protein encapsulated within the hydrogel matrix remain folded in its native form for a successful protein delivery application. The pores within the hydrogel network could be a suitable space to encapsulate and protect the protein molecules from any external denaturant. In one of their pioneering works, Zhang et al. reported the encapsulation and release kinetics of various proteins (lysozyme, trypsin inhibitor, BSA, and IgG) from RADA peptide (Ac-(RADA)_4_-CONH_2_) hydrogel (Koutsopoulos et al., 2009).

The advent of drug resistance amongst various strains of microbial as well as neoplastic cells have rendered majority of the conventional therapeutic intervention procedures futile and the modern day medication techniques invariably employ combination therapies, i.e., the administration of multiple drugs that act through distinctly different mechanisms and synergistically restrain the proliferation of diseased cells. Moreover, a time-gated dosage of drugs in a sequential manner has been observed to be highly potent against many cancer cell types and to this end, Fatouros et al. developed a dual drug delivery platform wherein the aqueous solubility of the loaded drugs primarily dictated their diffusion mediated release kinetics and helped in realising synergistic anticancer therapy against HSC-3, Human Tongue Squamous Carcinoma Cell Lines (Karavasili et al., 2019). The peptide gelator (Ac-(RADA)_4_-CONH_2_), was employed as a carrier for delivery of curcumin and doxorubicin in a controlled fashion. Doxorubicin, owing to its hydrophilic character was rapidly released from the hydrogel meshwork with 80% of the drug released within first 4 h of administration and complete drug release in 24 h. Curcumin, however, because of its hydrophobic nature, was retained in the hydrophobic domains of the peptide nanofibers and was steadily released over a period of 19 days (62% total drug release). *In-vitro* as well as *in-vivo* studies demonstrated that the initial burst release of Doxorubicin followed by a sustained release of curcumin resulted in a dose-dependent apoptotic response against HSC-3 cells. This study also highlights the importance of rationally selecting drug molecules with desirable physicochemical attributes to realise programmable and tuneable drug release kinetics.

The concept of time-gated combination therapy is extremely crucial in serious health issues like Ischemia-reperfusion (I/R)-induced organ injury wherein simple administration of anti-inflammatory agents is not sufficient as the tissues with I/R injury display poor recovery rates and are extremely susceptible to chronic fibrosis, eventually leading to permanent organ dysfunction. Liu et al. designed a self-assembling peptide, KLD2R (Ac-KLDLKLDLKLDLRR-CONH_2_), and heparin based hydrogel which could co-deliver TNF-α neutralizing antibody (anti-TNF-α) and hepatocyte growth factor (HGF) in a time-controlled fashion (Liu et al., 2020). The KLD2R hydrogel owing to its strong cationic nature (due to the presence of two arginine units), binds negatively charged heparin in its fibrillar network which in turn exhibits high affinity for HGF via molecular shape recognition mechanism. The anti-inflammatory agent, anti-TNF-α, however, is simply embedded in the gel matrix and thus, displayed faster diffusion mediated release kinetics (∼38 and ∼70% released at 48 and 120 h, respectively) at the site of injury.

Hartgerink et al. developed a singular drug delivery platform wherein orthogonal self-assembly of multidomain peptide (MDP) hydrogels entrapping liposomal drug carriers allowed for the bimodal delivery of growth factors (GFs) and cytokines (Wickremasinghe et al., 2014). The MDP used here, K(SL)_3_RG (SL)_3_KGRGDS, was tailored with a MMP2 sensitive LRG sequence and cell adhesive RGD sequence, and could effectively entrap multiple growth factors, well partitioned between the gel matrix and the embedded liposomes. Release studies using GFs/cytokines of diverse sizes and activities showed that the diffusion controlled release of cargo from the gel matrix displayed faster release kinetics as compared to degradation controlled release of bioactive factors from the gel entrapped liposomes. This enabled autonomous and temporally controlled release of multiple cargoes from the composite hydrogel and can be utilized for enhancing the efficacy of combination therapies.

**FIGURE 5:**
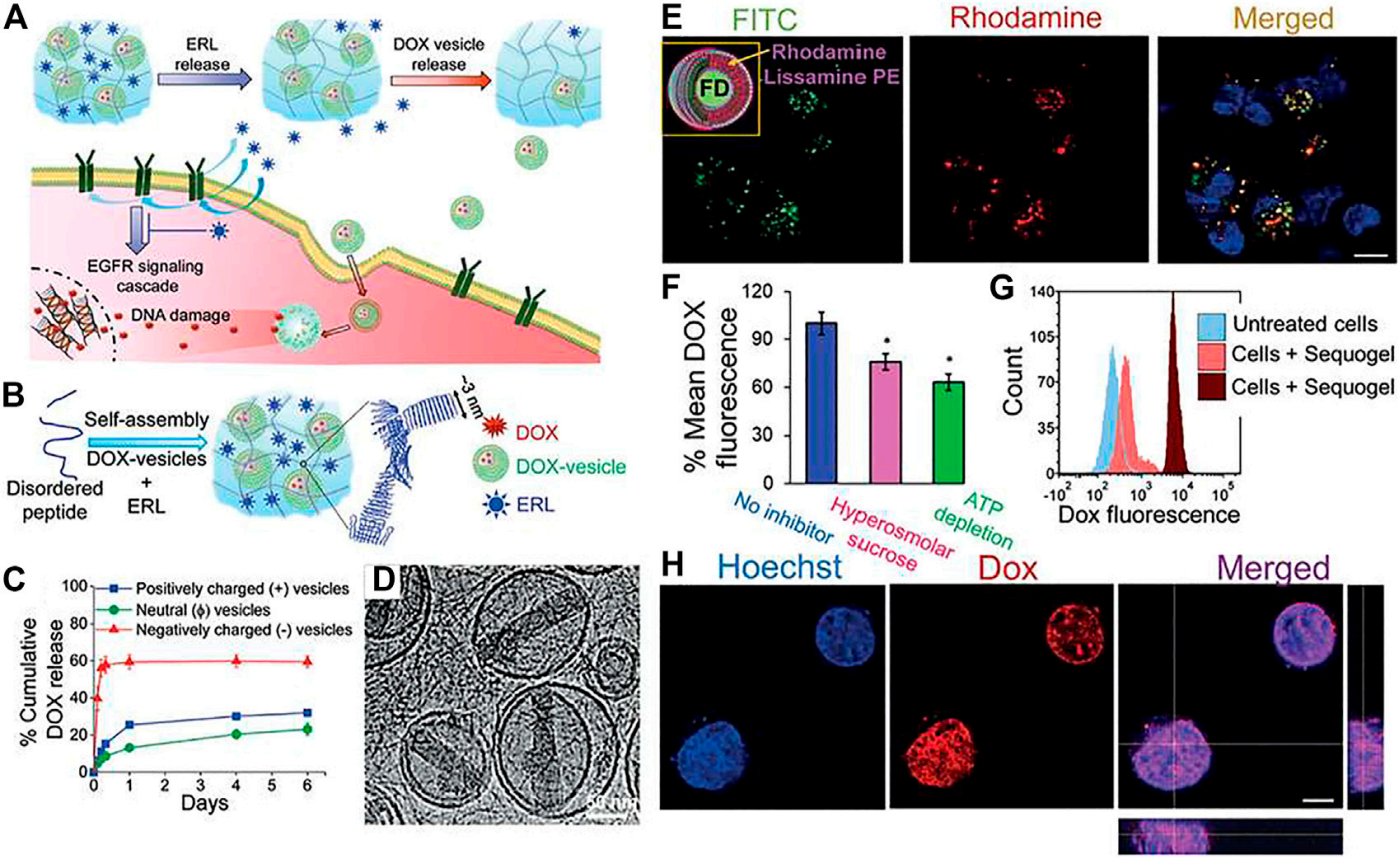
The time-staggered delivery of ERL and DOX-containing vesicles from Sequogel. **(A)** Schematic presentation of the time staggered delivery process. **(B)** Schematic diagram showing preparation of Sequogel consisting of MAX8 peptide in presence of ERL and DOX-loaded vesicles. **(C)** DOX releaseprofile from 1 wt% peptide gel containing negatively charged (POPS/PC/cholesterol), positively charged (DOTAP/PC/cholesterol), and neutral (PC/cholesterol) vesicles. **(D)** Cryo-TEM image of Sequogel containing DOX-loaded vesicles encapsulated within the peptide hydrogel. **(E)** a) LN229 cellular uptake and localization of labeled vesicles released from Sequogel. **(F)** Mechanism of cellular uptake of DOX-loaded vesicles released from Sequogel. Cells were incubated with Sequogel for 1 h with and without endocytic inhibitors. Mean fluorescence intensities of DOX under each inhibition condition were compared to those in absence of inhibitor to determine statistical significance, indicated by * for *p* < 0.05. **(G)** Histograms from flow cytometric detection of DOX internalization 4 and 16 h post-incubation with Sequogel. **(H)** Live-cell imaging of LN229 cells after addition of Sequogel containing DOX vesicles and ERL at 16 h post-incubation. Reproduced with permission (Majumder et al., 2018). Copyright 2018 Wiley-VCH.

A similar strategy was employed by Schneider et al. for time staggered sequential co-delivery of erlotinib (ERL, an EGFR inhibitor) and DOX (loaded inside vesicles) using an injectable multicompartment hydrogel delivery platform termed “Sequogel” incorporating MAX8 peptide (Haines-Butterick et al., 2007) as the hydrogel component and different charged/neutral liposomes as the DOX encapsulating modules (Figure 5) (Majumder et al., 2018) ERL alone displayed meagre apoptotic response against glioblastoma whereas DOX displayed only 21% apoptotic response when administered alone. However, owing to the ability of ERL to sensitize cancer cells towards apoptotic damage by DNA disrupting drugs like DOX, the two therapeutic agents when administered in a sequential manner using the “Sequogel” markedly enhanced the apoptotic extermination of cancer cells. The rapid release of ERL followed by sustained release of DOX from the neutral liposomal encapsulants displayed the best release kinetics and resulted in 40–59% apoptotic response at varying drug loading concentrations. Incorporating slow-eluting vesicular carriers in hydrogel matrix thus presents an attractive approach to realizing prolonged release of therapeutics in a time-delayed fashion.

### Wound Healing

Wound healing is an important biological process. It involves, replacement of injured tissues, restoring of the skin barrier’s function, and maintaining the internal homeostasis. Dressings (with or without any loaded drug) that can offer a moist environment conducive to healing is an ideal situation. For this reason, hydrogels and especially peptide based hydrogels are ideal candidates for wound healing process and a plethora of such examples are charted in recent literature. Wound healing by a peptide hydrogel can also be considered as a drug delivery system. However, as the application is topical, unlike internal localized delivery systems, the prerequisite of injectability is absent in this case.

Hauser et al. have reported several short peptide based wound healing systems in recent years (Seow et al., 2016). In one of their works, they have chosen two Cysteine containing peptides, LIVAGKC and LK_6_C, for the evaluation on mice model with full-thickness excision wounds. The disulphide linkages formed during the self-assembly process enhanced the mechanical properties of these hydrogels and the LK_6_C hydrogels could be handled easily with forceps during surgical procedures and used for wound healing purpose. Topical application of the LK_6_C hydrogel as a dressing accelerated re-epithelialization compared to controls and showed potential as a wound healing dressing.

For a burn wound, the dressing essentially needs to perform the following, 1) prevent infection, 2) maintain skin hydration to facilitate debridement of the necrotic tissue and 3) provide cues to enhance tissue regeneration. In one of their earlier reports, Hauser et al. have shown the superior effect of short peptide based hydrogels as a dressing for burn wounds over the commercially available Mepitel^®^, a silicone-coated polyamide net (Loo et al., 2014). Two non-toxic peptide (Ac-ILVAGK-NH_2_ and Ac-LIVAGK-NH_2_) hydrogels were used as dressings. Both the peptides showed excellent epithelial and dermal regeneration in the absence of exogenous growth factors and after 14 days, 86.2 and 92.9% wound closures were achieved respectively. In the same timeframe, Mepitel^®^ showed only 67.2% healing.

In order to rapidly control the inflammatory effect and accelerate wound healing, Zhong and co-workers recently developed a novel dual drug delivery system using a short peptide sequence, Nap-GFFKH (Cui et al., 2020). Bovine basic fibroblast growth factor (FGF-2) was constructed in fibrous alginate and encapsulated in the antibiotic-loaded peptide hydrogel. The hydrogel showed appropriate rheological behaviour for wound dressing. When applied for full-thickness excision wounds, the antibiotic gets released rapidly while FGF-2 released gradually within 7 days.

**FIGURE 6:**
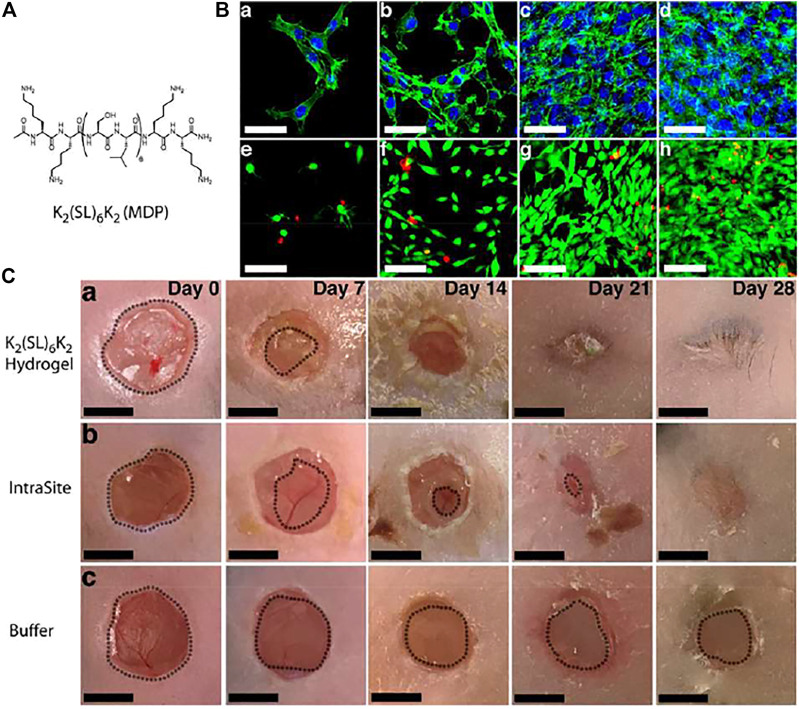
Application of peptide hydrogel for wound healing. **(A)** Chemical structure of the peptide hydrogelator (MDP) used for the study. **(B)** 3D cell culture of NIH-3T3 fibroblasts in MDP hydrogel. Images of encapsulated NIH-3T3 fibroblasts within the MDP hydrogel after (a) 3, (b) 7, (c) 10, and (d) 14 days and stained with Alexa488-phalloidin (green) and DAPI (blue) to visualize the actin cytoskeleton and nuclei, respectively. Scale bars = 50 μm. Calcein Am (green) and ethidium homodimer-1 (red) were used for live−dead staining of fibroblasts at (e) day 3, (f) day 7, (g) day 10, and (h) day 14. Scale bars = 100 μm. **(C)** Expedited wound closure in diabetic mice by MDP hydrogel. Images of wounds taken for (a) MDP hydrogel, (b) IntraSite, and (c) buffer to measure wound contraction and wound closure (outline) in ImageJ showing faster healing with MDP hydrogel. Scale bars = 5 mm. Reproduced with permission (Carrejo et al., 2018). Copywrite 2018 American Chemical Society.

Diabetic patients suffer from chronic wounds as diabetes often involves endothelial dysfunction. An integrin-binding prosurvival peptide derived from angiopoietin-1, QHREDGS was used as a therapeutic candidate for diabetic wounds. Application of chitosan–collagen hydrogel with immobilized QHREDGS in full-thickness wounds in diabetic mice showed significantly accelerated and enhanced wound closure compared with a clinically approved collagen wound dressing, peptide-free hydrogel, or blank wound controls (Xiao et al., 2016). The use of multidomain peptide (MDP) hydrogels could be beneficial for wound healing as these hydrogels undergo rapid cell infiltration and elicit a mild inflammatory response promoting angiogenesis. Hartgerink et al. applied a short MDP (K_2_(SL)_6_K_2_) hydrogel to diabetic mice with full-thickness wounds (Figure 6). The addition of the hydrogel allowed granulation tissue and re-epithelialization to beat wound contraction without additional growth factors/cells. The MDP hydrogel resulted in excellent recovery of wounds with thick granualation tissue, dense vascularization, innervation by the peripheral nervous system and regeneration of hair follicle (Carrejo et al., 2018). There are several other reports on the use of peptide hydrogels for wound healing purpose. The primary issue that one need to consider before using peptide based hydrogels as wound dressing is the mechanical property of the hydrogel. If the hydrogel is not sufficiently strong to hold its structure it is difficult to use it as a dressing and in such cases, composite hydrogels with biopolymers could be beneficial.

### Antimicrobial Peptide Hydrogels

Antimicrobial peptides (AMPs) are ubiquitously found in all living beings as a core component of the innate defence mechanism and are equipped with multifaceted properties and functionalities to protect the host organism from different types of pathogenic infestations (Zou et al., 2020). The bactericidal properties of AMPs, generally, roots from their structural features such as overall charge and secondary conformations, and majority of the naturally occurring AMPs are found to be amphipathic (i.e., bearing hydrophobic as well as hydrophilic peptide segments) and cationic in nature (i.e., rich in Arg and Lys amino acids). The selective toxicity of AMPs against microbes arises from the distinct lipid composition of the bacterial (prokaryotic) cell membranes which are largely composed of negatively charged phospholipids as compared to mammalian and plant (eukaryotic) cell membranes which exclusively contain electrically neutral zwitterionic phospholipids (Matsuzaki, 1999). Electrostatic interaction between cationic AMPs and anionic bacterial membranes leading to membrane permeabilization and disruption has been identified as the most common mechanism of antimicrobial action. Assimilating the structural attributes of AMPs into peptide hydrogels has been found to be advantageous owing to the close resemblance of peptide hydrogels to the natural extracellular matrix (ECM) and the ability to mould them into three dimensional (3D) biocompatible and cell/tissue adhesive scaffolds critical for topical biomedical applications.

In biological systems, the mechanical integrity of ECM is maintained by an enzyme, lysyl oxidase (LO), that catalyses the formation and repairing of ECM by inducing cross-linking of ECM proteins such as collagen and elastin via oxidation of lysine and hydroxylysine residues to reactive aldehydes. This inspired Lu and Xu et al. to develop a LO or plasma amine oxidase (PAO) mediated hydrogelation technique wherein an aqueous solution of an AMP, A_9_K_2_, underwent hydrogelation in the presence of LO/PAO (Bai et al., 2016). The amphiphilic AMP A_9_K_2_ assembles into micelles and short nanorods in aqueous medium and displays bactericidal effect via membrane permeabilization mechanism. The non-assembling precursors undergo inter as well as intramolecular crosslinking in the presence of LO in fetal bovine serum (FBS) or PAO and form long entangled fibrillar networks. The hydrogel exhibited marked antibacterial activity against both Gram-negative and Gram-positive strains on the gel surface as well as in the supernatant solution. Moreover, the hydrogel displayed negligible cytotoxicity against mammalian cells and selectively killed pathogenic cells only.

Unlike most cationic AMPs, Banerjee et al., designed an array of lysine and arginine-free cationic AMP amphiphiles [H_2_N-(CH_2_)_n_CONH-Phe-CONHC_12_ (P1-5, where n = 1–5, C_12_ = dodecyl), Table 3] which assemble into thermoreversible and thixotropic hydrogels in water (pH = 6.6) (Nandi et al., 2017). The hydrogels of P4 and P5 (with n = 4 and 5, respectively) displayed much higher mechanical strength as compared to other analogues owing to their higher hydrophobic character and demonstrated marked inhibitory effects against Gram-positive (*B. Subtilis* and *S. Aureus*) as well as Gram-negative (*E. coli*) bacteria. The remarkable antibacterial activity of P4 and P5 can be attributed to the ordered alignment of free amino groups along the surface of the fibrillar networks. Furthermore, the hydrogels exhibited proteolytic stability against proteolytic enzymes, proteinase K and chymotrypsin, and presented negligible haemolytic activity and cytotoxicity at their respective MICs.

As mentioned earlier, the secondary structural conformation of peptide gelators in a fibrillar entanglement markedly dictates the antimicrobial efficacy of the hydrogel and β-sheet structural motifs, particularly, are known to form transmembrane pores in bacterial membranes, subsequently leading to membrane perturbation, cell leakage and cell death. This led Ghosh et al., to design a β-sheet forming AMP hydrogelator, PA-NV, comprising of a β-sheet forming hexapeptide NAVSIQ in the middle, a triple lysine residue at the C-terminal to achieve the desired antibacterial activity and a palmitoyl group at the N-terminal to reinforce hydrogelation (Adak et al., 2019). The hydrogel effectively neutralized both Gram-positive (*S. aureus*) and Gram-negative (*E. coli*) bacterial strains. Also, it averted enzymatic digestion (proteinase K) and displayed non-haemolytic activity towards hRBCs and non-cytotoxicity against normal cells.

Although a multitude of antimicrobial peptides and drugs have been developed and discovered over the decades, the intensive use of antibiotics have led to the emergence of multidrug resistant microbes which are impervious to conventional antimicrobial therapeutics. One such notorious antibiotic-resistant bacteria is methicillin-resistant *Staphylococcus aureus* (MRSA), which is extremely contagious and is responsible for colossal mortality rate across the global. To this end, Tang et al. developed a novel peptide based co-assembled hydrogel that effectively and specifically captures and kills MRSA bacteria (Zhao et al., 2019). A 5:1 M combination of Fmoc-L-Phe and D-phenylalanine conjugated oligo (thiophene ethynylene) (OTE-D-Phe, Table 3) yielded a transparent self-supporting hydrogel (HG-2) composed of thick and rough fibrillar networks capable of trapping both Gram-positive (MRSA) as well as Gram-negative (*E. coli*) bacteria. However, HG-2 selectively killed MRSA over *E. coli* owing to its ability to intercalate specifically into the lipid domains of MRSA membrane. Also, HG-2 exhibited remarkable bactericidal activity and non-cytotoxicity when coated over a model surface and thus is an ideal candidate for clinical applications.

Besides electrical charge and structural conformation of AMP based hydrogels, another crucial parameter that determines the antimicrobial efficacy of AMP hydrogels is their mechanical rigidity and stiffness. Zhong et al. demonstrated the implications of structural and mechanical aspects of antimicrobial hydrogels over their bactericidal properties by taking the examples of three inherently antibacterial peptide hydrogelators, Fmoc-tryptophan (Fmoc-W), Fmoc-methionine (Fmoc-M), and Fmoc-tyrosine (Fmoc-Y) (Xie et al., 2020). Fmoc-M and Fmoc-Y hydrogels exhibited flexible and entangled nanofibrillar network whereas Fmoc-W was composed of tightly packed rigid and aligned nanofibers. Fmoc-W hydrogel displayed much pronounced antibacterial activity against Gram-positive bacteria as compared to the other two hydrogels which highlights the fact that rigid nanofibrils can adhere strongly with bacterial membranes and elicit better cytotoxic response against pathogenic species.

Silver based antimicrobial agents have also been found to be effective within tolerable limits owing to their ability to generate ROS and adhere to cytoplasmic membrane of bacteria. At high concentrations, however, silver ions pose toxicity to mammalian cells. To achieve controlled release of silver ions, Makhlynets et al., designed a nonapeptide, Ac-(3′-PyA)LRLRLRL (3′-PyA)-CONH_2_ (L9, Table 3), bearing unnatural amino acid, 3′-pyridyl alanine (3′-PyA) at the two termini for binding silver ions and alternate leucine and arginine residues in the middle for adopting β-sheet self-assembling conformation (D’Souza et al., 2020). L9 weakly gelates water in the absence of silver ions. However, addition of 1 eqv. Ag(I) confers a strong hydrogel with 15-fold higher storage modulus. The 3′-pyridyl alanine group shows stronger binding with Ag(I) ions as compared to histidine and cysteine and the hydrogel releases Ag(I) in a sustained manner, thereby precluding any toxicity to normal cells. The hydrogel effectively inhibited the growth of Gram-negative bacteria (*E. coli*) through the synergistic effect of silver ions and arginine residues. Further, the pH independent release kinetics of the hydrogel allows operation even at reduced pH values often encountered in healing wounds.

### Tissue Engineering

Tissue engineering is one of the most important scientific advancement in the field of biomedical science and engineering that happened in last couple of decades. For a successful tissue engineering, a scaffold that mimics the natural extracellular matrix (ECM) is artificially created where cells are provided with environment appropriate for regeneration, induction, and development of neo-tissue. Peptide based hydrogel network could be an attractive choice as artificial ECM as they possess 3D network where cells can adhere and proliferate as a microenvironment is provided to the cells similar to that they encounter *in vivo*. Two detailed reports on peptide based hydrogels for tissue engineering application have recently been published separately by Zhang et al. and Ding et al. and can be used as ready references (Ding et al., 2020; Yang et al., 2020).

One of the most studied peptide hydrogel for 3D nanofibrous scaffold supporting cell differentiation is the RADA16 peptide family. These ionic peptides are able to form stable β-sheets and self-assemble spontaneously into cross-linked network structures, which could mimic the ECM of tissue cells. For example, three different peptide hydrogels were used to create a 3D cell environment for human adipose stem cells through C-terminal functionalization of RADA16 peptide with biologically active peptide sequences (SKP, FHR, PRGD, Table 3) (Liu et al., 2013).

Bone is amongst the most important type of tissues that have been subjected to tissue engineering. Bone tissue engineering (BTE) process requires seeding of stem cells, osteoblast or chondrocytes into a 3D scaffold. One of the prerequisites of BTE is the capability of biomineralization and appropriate functionality on the scaffold is required for this purpose. Optimum mechanical strength is also essential for a successful BTE. RADA, FEFEFKFK based hydrogels have successfully been tested on several occasions for this purpose (Misawa et al., 2006; Castillo Diaz et al., 2016). Stupp, reported the biominerilization of hydroxyapatite (HA) on a designed peptide amphiphile based system (Hartgerink et al., 2001). Composite hydrogel of extremely short peptides like QK/RGD have recently been reported that could enhance angiogenesis and osteogenesis simultaneously (Hung et al., 2020). We have very recently shown that the composite hydrogel formed by PyKC (59) and hyaluronic acid can be a very good candidate for bone tissue regeneration (Chowdhuri et al., 2021).

**FIGURE 7:**
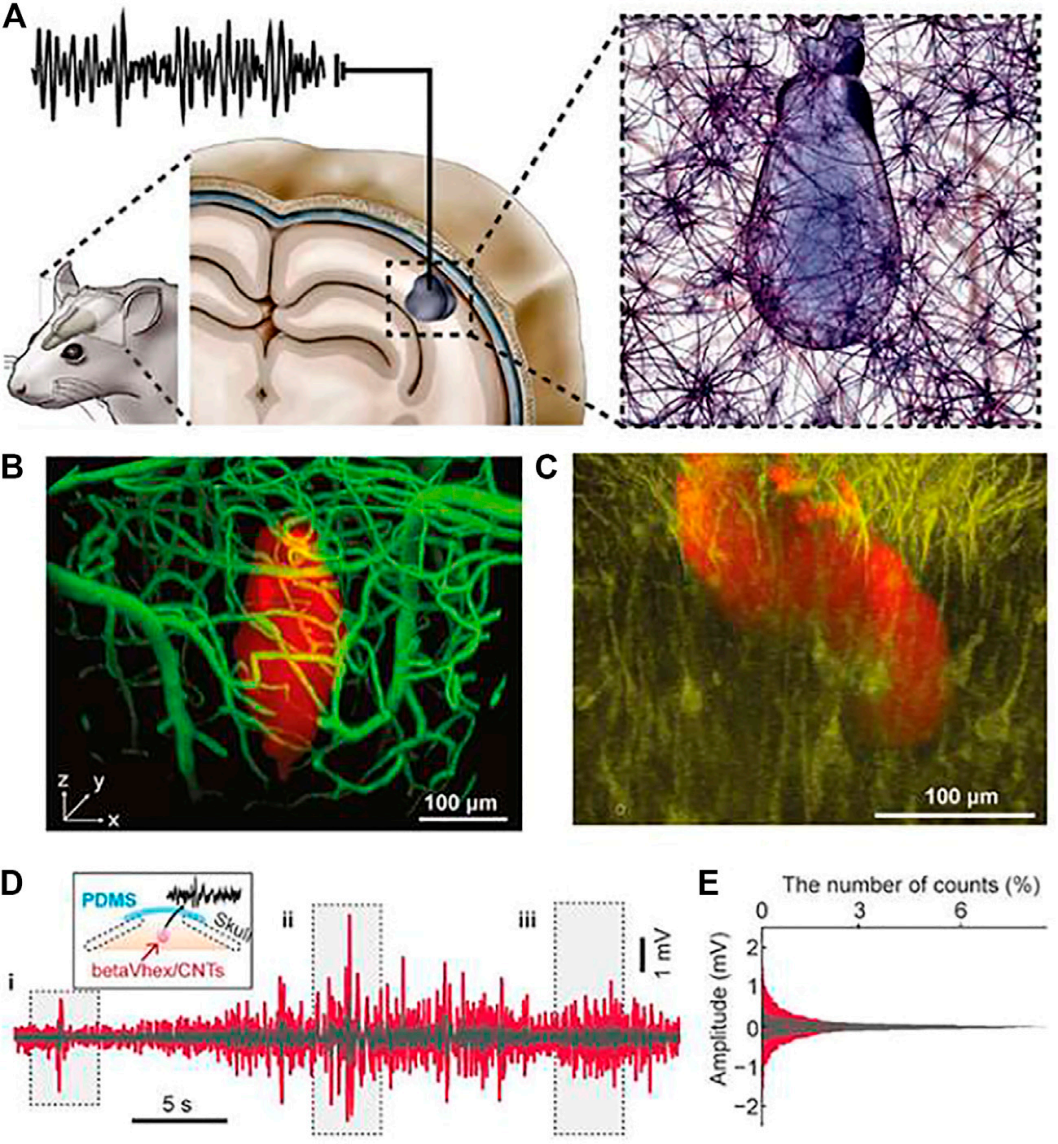
Peptide hydrogel for neurone repair. **(A)** Schematic presentation of the hydrogel injected into the mouse brain cortex and interacting with neurons. **(B)** Image (side view) of CNT-peptide composite hydrogel injected within the brain cortex and the brain vasculature visualized by FITC-dextran injection. **(C)** Image (side view) of neuronal structure (Thy1-GCaMP6f mouse) and the injected hydrogel. **(D)** LFP (local field potential) recording; red, LFP signals measured with the hydrogel; gray, those measured with bare electrodes inserted 1 mm apart. The inset figure is a schematic illustration of LFP measurements with the CNT-peptide composite hydrogels through the PDMS. **(E)** Distribution of the LFP signal amplitudes shown in **(D)**. Reproduced with permission (Nam et al., 2020). Copyright 2020 American Chemical Society.

Neural tissue engineering is another challenging task owing to the complexity of the neural system. Several successful attempts have been made to construct peptide-hydrogel based systems to heal neural injuries. Recently, a composite hydrogel of a peptide (KVKEVFFVKEVFFVKEVY), and carbon nanotube (CNTs) is reported that could augment the neural signal due to the presence of CNTs at the time of seizers in the epidural tissue (Figure 7) (Nam et al., 2020) A multihierarchical structure is formed by this non-biodegradable β-peptide in presence of conductive CNTs, creating a three-dimensional electrical network. Seamless integration of the composite hydrogel in brain tissue resulted in enhancement of the epidural and intracortical neural signals especially in the high frequency range. Amongst other tissues or organ repairing applications that have been reported with the help of peptide hydrogels are skin, cardiac, ligament, cartilage and cochlea to name a few (Ding et al., 2020). Though a considerable effort has so far been made in this particular area, there are many scientific and technological challenges to overcome. Insufficient understanding of the optimal mechanical property that is required for proper signalling between the cells as well as biocompatibility for the neo-cells are some of the important concerns that need to be addressed in order to use peptide based hydrogels for applicable tissue-engineering processes.

### 3D Biofabrication

Biofabrication is a sub-section of tissue engineering and the recent development in this area demands a separate discussion. 3D Biofabrication can be divided into two categories, 3D bioprinting and electrospinning.

**FIGURE 8:**
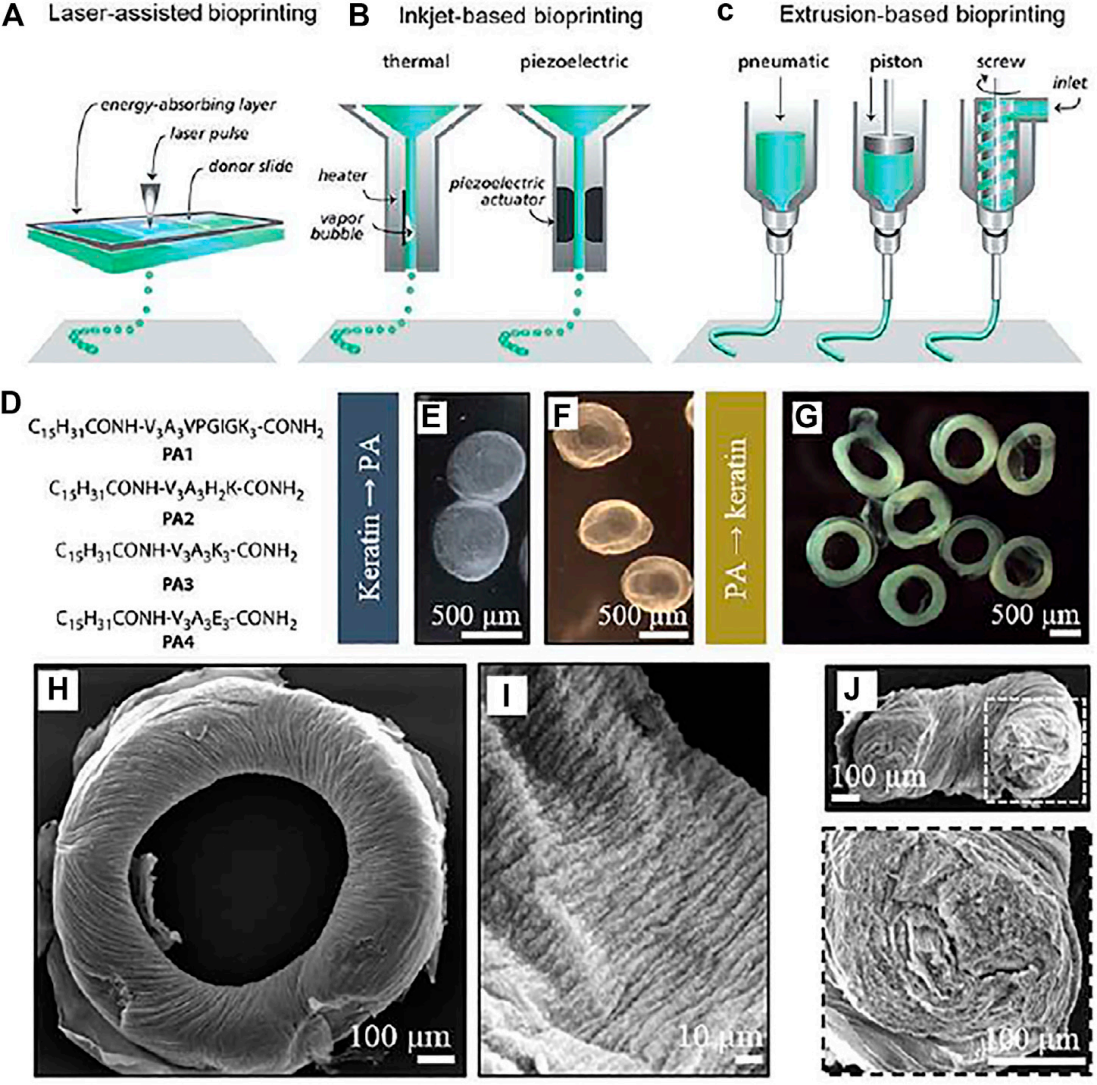
Use of peptide based hydrogels in 3D bioprinting. **(A–C)** Three different approaches for biofabrication involving the use of hydrogels in the form of “bioink”. D) Sequences of peptide amphiphile based hydrogels used for 3D printing. Images of E) biconcave gels made with keratin (20 mg ml^−1^) and **(F)** keratin (10 mg ml^−1^) jetted into PA1 (10 mg ml^−1^) and **(G)** of toroidal gels made with PA1 (10 mg ml^−1^) jetted into keratin (10 mg ml^−1^). **(H)** SEM image of one toroidal gel (500 μm) and **(I)** zooming in on the surface architecture of a toroidal gel, and **(J)** a cross-section of a PA1/keratin hydrogel and an enlargement of the right hand cross-section. **(A–C)** Reproduced with permission (Malda et al., 2013). Copyright 2013 WILEY VCH. **(D–J)** Reproduced with permission (Hedegaard et al., 2018). Copyright 2018 WILEY VCH.

3D bioprinting is a technological advancement where biological constructs of millimetre to centimetre size are printed. The technique is utilized to prepare realistic and transplantable 3D tissue or organs. The process of bioprinting can be divided into three basic steps: 1) Pre-processing—preparation of the bio-ink and preparation of the “blueprint” (computer-aided design, CAD); 2) Processing—printing of the 3D structure, and 3) Post-processing—the culture of the printed construct in bioreactor. The 3D bioprinting technologies can primarily be classified into three categories based on their working principles, inkjet-based, laser-assisted, and extrusion based bioprinting (Figure 8). Detailed analyses of these technologies have been reported in various excellent reviews (Malda et al., 2013; Pati et al., 2016).

The use of peptide based hydrogel as a bioink is advantageous as they can easily be fine-tuned in terms of physicochemical properties. Moreover, because of the presence of peptides as the raw material, possibility of rejection by cells is much lesser than that for synthetic polymeric systems. One of the first synthetic peptide based bioink was reported by Hauser and co-workers. In their report, they used a short peptide (Ac-ILVAGK-NH_2_) solution through fine-gauged needles (Loo et al., 2015). During this process, the peptides were aligned, and the structural integrity was achieved as the viscosity and rigidity increased. Over 6 days post-encapsulation on the scaffold, growth and elongation was observed for human mesenchymal stem cells (hMSCs). A commercial peptide-based hydrogel (PeptiGelDesign Ltd.) has recently been used to print well-defined hydrogel construct with variable rigidity and enhanced structural integrity (Raphael et al., 2017). In a recent report, Mata et al. reported the co-assembly of peptide amphiphiles with a range of extracellular matrix (ECM) proteins and biomolecules including fibronectin, collagen, keratin, ELPs, and hyaluronic acid during printing in cell diluent conditions. NIH-3T3 and adipose derived stem cells are bioprinted within complex structures using these composites (Hedegaard et al., 2018). Although the use of peptide based hydrogels have successfully been demonstrated by various groups, there is still a long way to go before these peptide based systems can be used for real therapeutic applications. One major challenge in this direction is to develop a standardized protocol and test on the printability of such soft materials.

## Conclusion and Outlook

The last two decades have experienced a significant advancement in the area of supramolecular chemistry, both in terms of fundamental understanding as well as applications. Peptide based hydrogels are no exception and extensive effort have been made toward their applications as therapeutics. Despite of this progress, there is much room for growth in this area. There remains a critical need to understand the major factors involved in the self-aggregation of peptides to construct higher order assemblies and to demonstrate rational design criteria to form reproducible smart hydrogels with definite structure and properties. Rational design with foreseeable structures and properties is essential for the progress in this area. Bio-medical applications are complex and thus the material to be used need to satisfy many parameters where many of these materials often fail. It is important to identify those parameters as well as the reasons behind any failure. Unfortunately, the scientific community, in general, does not report the failures. That itself make things even more challenging.

Before closing this review, we want to mention that, though majority of the reports come with proper understanding and detailed analyses, a section of literature raises some critical issues which need considerable attention of the peers in this field. An important issue in this regard is the use of hydrogels showing drug entrapment and release without analysing whether they are injectable or not. It is certain that unless the hydrogels are injectable or follow the *in-situ* formation protocol, their application as a drug delivery vehicle remain restricted to topical use only. However, by encapsulating a drug and releasing it in bulk aqueous medium (with or without any stimuli) does not really make them suitable drug delivery vehicles. At the most, these experiments can be termed as “model drug release” studies. In this regard, the *in-vitro* drug release experimental protocols are also erroneous on many occasions. Typically, the drug loaded hydrogels are prepared in a vial and on top of the hydrogel, the required buffer is added. The supernatant buffer is checked for the released drug in a time dependant manner. It is understandable that in this widely used methodology, not all the hydrogel surface but the top of the hydrogel is in contact with the buffer. The surface area of the top surface depends on the inner diameter of the vial used. Thus, whatever the extent of release obtained from these studies are erroneous as the surface areas, in general, are not taken into consideration while calculating the release profile. Biological systems are complex and using any artificial material for therapeutic applications require proper and detailed investigation. The *in-vivo* use of the hydrogels requires extensive studies on the degradation and excretion pathways of the hydrogelator. Moreover, interaction with other molecules present in the living systems also need to be analysed before such systems are used for real applications. We are hopeful that the guideline provided in this review will be helpful to construct newer materials with advanced properties and more effective therapeutic applications in future.
